# Lung Cancer Gene Regulatory Network of Transcription Factors Related to the Hallmarks of Cancer

**DOI:** 10.3390/cimb45010029

**Published:** 2023-01-05

**Authors:** Beatriz Andrea Otálora-Otálora, Liliana López-Kleine, Adriana Rojas

**Affiliations:** 1 Grupo de Investigación INPAC, Unidad de Investigación, Fundación Universitaria Sanitas, Bogotá 110131, Colombia; 2 Facultad de Medicina, Universidad Nacional de Colombia, Bogotá 11001, Colombia; 3 Departamento de Estadística, Universidad Nacional de Colombia, Bogotá 11001, Colombia; 4 Facultad de Medicina, Instituto de Genética Humana, Pontificia Universidad Javeriana, Bogotá 110211, Colombia

**Keywords:** lung cancer, pulmonary arterial hypertension, differentially expressed genes, transcription factors, co-expression networks, common connection patterns, gene regulatory network, coregulatory network, fibration symmetries, transcriptional regulatory network, lung cancer biomarkers, patient’s survival, transcriptomics and bioinformatics

## Abstract

The transcriptomic analysis of microarray and RNA-Seq datasets followed our own bioinformatic pipeline to identify a transcriptional regulatory network of lung cancer. Twenty-six transcription factors are dysregulated and co-expressed in most of the lung cancer and pulmonary arterial hypertension datasets, which makes them the most frequently dysregulated transcription factors. Co-expression, gene regulatory, coregulatory, and transcriptional regulatory networks, along with fibration symmetries, were constructed to identify common connection patterns, alignments, main regulators, and target genes in order to analyze transcription factor complex formation, as well as its synchronized co-expression patterns in every type of lung cancer. The regulatory function of the most frequently dysregulated transcription factors over lung cancer deregulated genes was validated with ChEA3 enrichment analysis. A Kaplan–Meier plotter analysis linked the dysregulation of the top transcription factors with lung cancer patients’ survival. Our results indicate that lung cancer has unique and common deregulated genes and transcription factors with pulmonary arterial hypertension, co-expressed and regulated in a coordinated and cooperative manner by the transcriptional regulatory network that might be associated with critical biological processes and signaling pathways related to the acquisition of the hallmarks of cancer, making them potentially relevant tumor biomarkers for lung cancer early diagnosis and targets for the development of personalized therapies against lung cancer.

## 1. Introduction

Lung cancer is an unrestrained tumor cell growth that can invade and affect other tissues [1]. Lung cancer caused 11.4% of the deaths associated with cancer around the world in 2020 [2]. Non-small-cell lung cancer (NSCLC) is the most frequent type represented by 85% of the cases, and small-cell cancer (SCLC) is the less frequent with 15% of the total cases [3]. The NSCLC histological subtype adenocarcinoma (LAC) is responsible for around 40% of the cases, squamous cell carcinoma (SCC) is around 30%, while large-cell carcinoma is very rare, at approximately 5–10% [4]. The potential treatment of lung cancer is limited by the diagnosis based on the appearance of symptoms only in the late stages of the disease [1]. Multiple genetic risk factors have been identified, such as mutations, gene amplifications, deletions, and fusion genes, which have been associated with an increase in the susceptibility to developing lung cancer [5]. However, no pharmacological treatment has shown important changes in the tumoral phenotype of all patients. In this study, it is proposed that the lack of effective lung cancer biomarkers is associated with the fact that its identification has not reached the complete molecular and cellular variability in the tumor environment of each individual and population of individuals, and the group of important biomarkers have not been identified within the significant number of deregulated genes identified by genetic studies [6].

The transcriptomic studies that look for the complete gene expression scenario, such as microarrays and RNA-Seq, have provided biological information about dysregulated genes involved in cancer [7]. We have already performed a deeper analysis of microarray studies used with a different bioinformatic pipeline, taking advantage of the huge amount of knowledge that could be generated with this technology, and therefore, increasing the understanding of cellular processes related to the early stages of complex diseases such as lung cancer [6]. Our previous joint transcriptomic analysis allowed for the identification of possible key biomarkers for early detection and the future development of treatments against NSCLC. In the present work, we also want to show how the re-analysis of gene expression data available in multiple databases can be used to obtain new knowledge and propose new biological hypotheses based in a deeper and more focused bioinformatic pipeline, in order to find a potential gene network of biomarkers associated with the regulation of the unique tumoral functions in lung cancer. 

The selection microarray and RNA-Seq datasets of all possible types and subtypes of lung cancer will reach all deregulated genes and transcription factors specific to lung cancer, regardless of their histopathologic classification. Moreover, the construction of co-expression, gene regulatory, coregulatory, and transcriptional regulatory networks can allow us to identify synchronized biological clocks of deregulated genes co-expressed in lung cancer, which are controlled by transcription factors inside the fibration symmetries of the gene networks [8], leading to a better understanding of the oncogenic cellular function embedded in their interactions [9]. Increased pulmonary arterial pressure has been associated with the response to treatment in patients with lung cancer [10]. Moreover, the possible association between the pulmonary arterial hypertension dataset (PAH) and lung cancer [11], through different physio-pathogenic mechanisms, has previously been suggested in several studies, with evidence that other pulmonary diseases share processes characteristic of lung cancer that point to a possible causal association in the acquisition of the hallmarks of cancer [6]. Therefore, it is important to take this comorbidity into account when identifying transcriptomic risk factors for developing lung cancer. 

Our bioinformatic pipeline seeks to identify a unique transcriptional regulatory metafirm of lung cancer transcription factors associated with the acquisition of the hallmarks of cancer during the lung tumoral process. Moreover, the lung cancer metafirm can be characterized by the formation of functional blocks of genes co-expressed in lung cancer that are regulated by a group of transcription factors important for the regulation of gene expression during the acquisition of the hallmarks of cancer; therefore, they may be interesting candidates that could be used as biomarkers for the development of diagnostic tools and specific treatments against lung cancer.

## 2. Materials and Methods

All datasets are case–control studies, measuring the gene expression (microarrays and RNA-Seq) of lung tumoral cells and adjacent non-tumoral lung cells using high-throughput sequencing (Table 1), which represent lung cancer pathological types (NSCLC and SCLC) and subtypes (squamous lung cancer and lung adenocarcinoma) from different populations. The differentially expressed genes (DEGs) list of the six microarray and four RNA-Seq datasets from our previous transcriptomic analyses in three types of cancer [12], along with the pulmonary arterial hypertension dataset (PAH) from our previous joint transcriptomic analysis (Table S1) [6], were compared to identify all deregulated genes in at least eight lung cancer datasets. This was in order to identify the most frequent lung cancer (LC) DEGs and highlight among them the most frequently dysregulated transcription factors (TFs) in lung cancer and PAH. Therefore, the most frequent DEGs and TFs are the dysregulated genes present in at least eight of the ten lung cancer datasets. The fold change was previously calculated for a given gene as the difference between the expression profile in cases versus controls, and the value was averaged across all datasets, such that genes with fold change values greater than one are upregulated, while those with fold change values less than one are downregulated [13]. The gene expression of the most frequent DEGs was validated with all TCGA gene expression studies on squamous cell carcinoma (SCC) and lung adenocarcinoma (LAC), available at The University of ALabama at Birmingham CANcer data analysis Portal (UALCAN), United States (http://ualcan.path.uab.edu/analysis.html accessed on 10 January 2023). The DAVID (Version 2021) functional and enrichment analyses with the official gene symbol of the most frequent LC DEGs identified those related to biological processes and signaling pathways that might be related to the acquisition of the hallmarks of cancer, through an enrichment analysis of gene ontology, signaling pathways, and gene–disease association terms [14]. The gene expression correlation of the most frequently dysregulated TFs in NSCLC (GSE19804) and SCLC (GSE108055) was analyzed with the R package corrplot (Version 0.92) [15]. The visualization method of the correlation matrix used was “color”, the ordering method of the correlation matrix was the hierarchical clustering “hclust”, the number of rectangles drawn on the graph according to the hierarchical cluster or addrect was “two”, and the colorRampPalette was "blue", "white", and "red". 

Co-expression networks were created for every dataset with a normalized gene expression matrix of the most frequent LC DEGs using the Pearson correlation coefficient, with a threshold of 0.7. The co-expression layers were compared to identify intersection subnetworks or common connectivity patterns (CCPs) with the Coexnet library (Version 1.15.0) in R, and to determine common elements or biomarkers among the co-expression networks [16], as well as the most frequent alignments with Gedevo, as those with the lowest graph editing cost, with a score representing the degree of similarity of each pair of nodes, or statistically significant alignments with a median above 0.5 [17]. Every CCP was constructed using the igraph package (Version 1.3.5) in R [18] and was analyzed with iRegulon application (Version 1.3) of Cytoscape (Version 3.9.1) [19,20] to identify a regulator or TF for every subnetwork, using motif and track discovery in proximal and distal sequences, around ten thousand candidate motifs or position weight matrices, gene rankings according to the highest ChIP peak within the regulatory space with over one thousand ChIP-Seq tracks, and Reactome FIViz application (Version 8.0.4) to perform a signaling pathway enrichment analysis, accessing the Reactome pathways stored database [21].

The most frequent LC DEGs list was then divided into DEGs found only in eight lung cancer datasets, and those that were found in eight lung cancer datasets and PAH, and each list was analyzed with the DAVID tool in order to identify biological process and signaling pathways related to the acquisition of hallmarks of cancer [14]. Gene regulatory networks (GRNs) of the most frequent DEGs and TFs were inferred with CoRegNet (Version 1.32.0), based on a hybrid version of LICORN, which combines both a discrete and a statistical model with an emphasis on regulator cooperativity [22]. This was performed for the two types of lung cancer—NSCLC (GSE19804) and SCLC (GSE108055)—in order to identify the most frequent DEG targets of the most frequently dysregulated TFs, related only to lung cancer (LC) and lung cancer along PAH (LC-PAH). The most frequent target DEGs of every TF were analyzed with the DAVID functional and enrichment analysis [14]. Then, GRNs were divided by TF targets related to the acquisition of every hallmark of cancer. ChIP-X Enrichment Analysis 3 (ChEA3 Version 3) performed a TF enrichment analysis (TFEA) in the lists of the most frequent LC DEGs, according to ChIP-seq experiments from ENCODE, ReMap, and individual publications; TF co-expression based on RNA-seq studies from GTEx and ARCHS4; co-occurrence of TFs with other genes performed by Enrichr; and genome-wide gene expression resulting from TF silencing experiments, in order to validate the target genes of the most frequently dysregulated transcription factors [23].

The coregulatory networks of most frequently dysregulated transcription factors were also inferred with CoRegNet, to find the coregulators in the GRNs or possible interactions at the protein level of the most frequently dysregulated TFs to accomplish its regulatory function. The R library “Fibration symmetries’’ (Version 1.1) was used to identify fibers or functional/biological blocks formed by the most frequently dysregulated TFs in every GRN [9], with the get.building.blocks function, and analyze the molecular functional synchronization of the lung cancer transcriptional network related to the biological processes and oncogenic signaling pathways involved in the acquisition of the hallmarks of cancer. 

**Figure 1:**
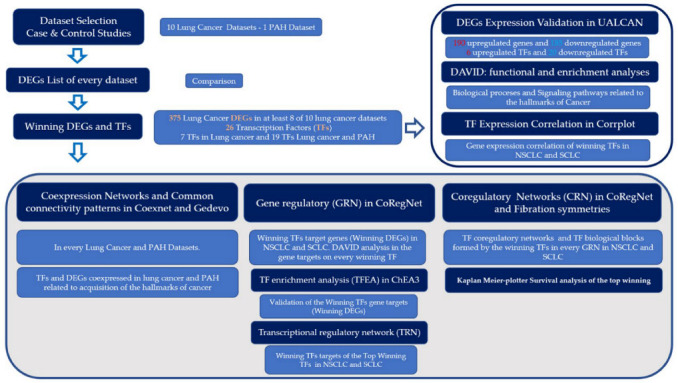
Bioinformatic pipeline for the construction of a lung cancer gene regulatory network of transcription factors related to the hallmarks of cancer.

A transcriptional regulatory network (TRN) was constructed to analyze if the top deregulated TFs might be able to recognize specific DNA sequences to control the expression of other frequently dysregulated TFs, with one hundred permutations in the RTN (Version 2.18.0) (Reconstruction of Transcriptional regulatory Networks and analysis of regulons library of R) [24]. The online Kaplan–Meier plotter tool (http://kmplot.com/analysis/index.php?p=service&cancer=lung accessed on 10 January 2023) was used to perform a meta-analysis-based discovery and validation of the top deregulated TFs identified with the transcriptomic analysis of lung cancer, using default settings and all probe sets available which include several lung cancer datasets from GEO, EGA, and TCGA. This revealed a correlation between the expression of the genes (mRNA, miRNA, protein) and patients’ survival, through the calculation of a hazard ratio of 95% confidence intervals and a log rank P-value [25]. The first stage of the bioinformatic analysis identified the most frequent lung cancer DEGs and TFs, validated their gene expression levels, found the related biological functions and signaling pathways, and performed a correlation analysis of the TF mRNA expression. The second stage started with a co-expression analysis of the most frequent DEGs and TFs, followed by a gene regulatory analysis to identify the most frequent DEG targets of the most frequent deregulated TFs, followed by a validation of the most frequent deregulated gene targets of the TFs. Then, a transcriptional regulatory network identified the most frequent deregulated TFs that can be regulated out of the top seven deregulated TFs. Finally, a coregulatory analysis was performed to identify the protein–protein complexes and functional blocks that can be formed by the most frequent deregulated TFs to accomplish its regulatory function during the acquisition of the hallmarks of cancer (Figure 1). The analysis was performed following our own bioinformatic pipeline with several packages in R (Table S2). 

## 3. Results

### 3.1. Co-Expression Networks Analysis

**Figure 2:**
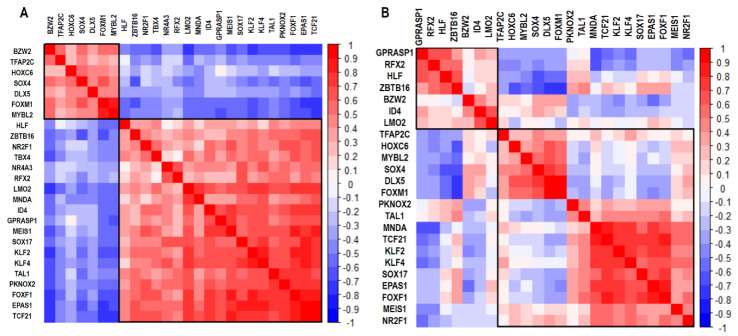
Correloplot of the mRNA expression correlation of the most frequently dysregulated transcription factors in every lung cancer type: (**A)** NSCLC and (**B)** SCLC. In blue are the negatively correlated TFs, and in red are the positively correlated TFs.

**Figure 3:**
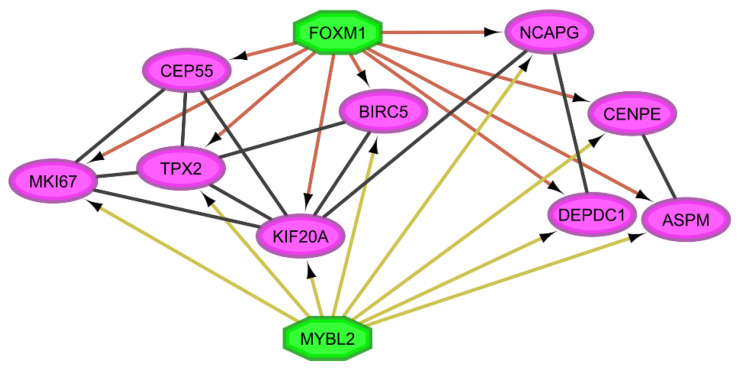
Common connection pattern (CCP) of all lung cancer co-expression networks and pulmonary arterial hypertension co-expression networks. In green are the two main regulators of the genes (magenta) in the CCP.

On average, there are 140 upregulated or downregulated genes in at least eight lung cancer datasets, and every lung disease dataset, except for PAH, has an average of 6500 deregulated genes (Table S1) [6,12]. The UALCAN analysis validated the expression of all 190 upregulated genes and 185 downregulated genes (Table S3). There are twenty-six deregulated TFs in at least eight of the ten lung cancer datasets, of which nineteen are also deregulated in PAH, and seven are only deregulated in lung cancer (Table 2). The gene expression correlation is positive and negative between different groups of the most frequently dysregulated TFs in the two types of lung cancer: NSCLC (Figure 2A) and SCLC (Figure 2B). All possible comparisons between co-expression networks of lung cancer and PAH have CCPs, which can be regulated by two top deregulated TFs, FOXM1 and MYBL2, according to the iRegulon analysis (Table 3). All co-expression networks have a CCP in common, and FOXM1 can regulate all nine genes, while MYBL2 regulates all except CEP55 (Figure 3). 

The CCPs formed between lung cancer and PAH have FOXM1 and FOXF1 co-expressed with the most frequent DEGs in microarrays and RNA-Seq datasets, respectively. The CCP formed between lung cancer co-expression networks of microarray datasets have TCF21, FOXM1, and MYBL2, while the CCP of lung cancer RNA-Seq datasets have TCF21, FOXM1, FOXF1, SOX17, TAL1, LMO2, KLF2, and TBX4 co-expressed with some of the most frequent LC DEGs. Moreover, P53, integrin-mediated, and Wnt signaling pathways have some nodes involved in LC-PAH CCPs, and ATR, FoxO, P53, and BMP signaling pathways have some nodes involved in LC CCPs (Table S4). Coexnet CCPs are related to multiple cell cycle and biological functions that might be related to the acquisition of hallmarks of cancer such as sustained angiogenesis, evading apoptosis, limitless replicative potential, activating invasion and metastasis, self-sufficiency of growth signals, insensitivity of anti-growth signals, and sustaining proliferative signaling (Table S4). 

The comparison of the LC co-expression networks with Gedevo identified eight alignments that appeared in at least 40% of the possible alignments, all of them also appear in the Coexnet CCPs, and four are statistically significant (median >0.5) (Table 4). ASPM and KIF20A are in Gedevo alignments and the CCP of all datasets (Figure 3), but only ASPM has a statistically significant alignment. Gedevo alignments are also related to multiple cell cycle functions, and some of them show evidence of dysregulation in cancer (ASPM, CENPF, TOP2A, and TPX2) and the acquisition of two hallmarks of cancer: sustaining proliferative signaling and evading apoptosis (Table S4).

### 3.2. Gene Regulatory Networks Analysis

**Figure 4:**
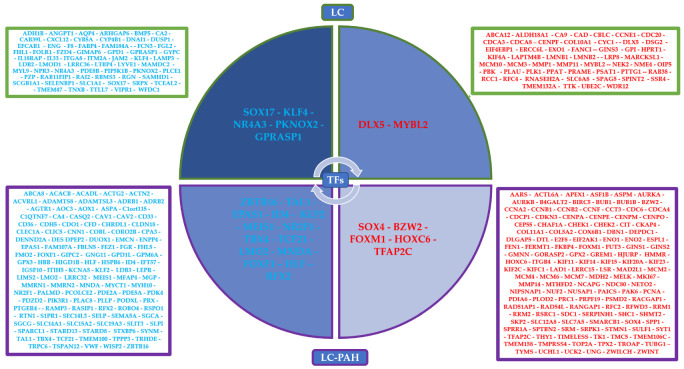
Cycle matrix with the winning transcription factors (TFs) and most frequently dysregulated differentially expressed genes (DEGs) inside the squares. Downregulated TFs and DEGs are in blue, and upregulated TFs and DEGs are in red. Those unique to lung cancer (LC) are in a green outline, and those shared by lung cancer and pulmonary arterial hypertension (LC-PAH) are in a purple outline.

In the 375 most frequent DEGs, there are 257 that are also deregulated in PAH, and 117 are deregulated only in lung cancer (Table S1) (Figure 4). According to the DAVID analysis, 92 common DEGs of lung cancer and PAH have experimental cancer-related evidence; moreover, there are DEGs related to the positive regulation of transcription, angiogenesis, cell division, growth, adhesion, differentiation, proliferation, migration, senescence, and apoptosis, as well as Wnt, BMP, insulin, metabolic, FoxO, and p53 signaling pathways, and among all of them are twelve of the most frequent TFs (SOX4, TAL1, FOXM1, KLF2, MEIS1, TBX4, EPAS1, ZBTB16, ID4, NR2F1, TFAP2C, and FOXF1). Furthermore, thirty-eight unique LC DEGs have experimental evidence of their association with cancer and DEGs related to the positive regulation of gene expression, cell division, growth, adhesion, proliferation, and differentiation, as well as TGF-𝛽, metabolic, and BMP signaling pathways, and among all of them are five of the most frequent TFs (SOX17, NR4A3, MYBL2, DLX5, and KLF4) (Table S1). 

The NSCLC and SCLC GRNs were made with the most frequent LC DEGs dysregulated in eight lung cancer datasets, and the most frequent LC DEGS dysregulated in eight lung cancer datasets and PAH, considering the most frequently dysregulated TFs (Table 2). The GRN analysis identified the top target DEGs dysregulated in lung cancer datasets and PAH of the most frequently dysregulated TFs deregulated in lung cancer and PAH, the top DEG targets dysregulated only in lung cancer datasets related to the most frequently dysregulated TFs dysregulated in lung cancer and PAH, and the most frequently dysregulated TFs only in lung cancer (Table 5). The most frequent LC DEG targets of the most frequently dysregulated TFs are related to multiple cell cycle and biological functions that might be related to the acquisition of hallmarks of cancer such as sustained angiogenesis, evading apoptosis, limitless replicative potential, self-sufficiency of growth signals, insensitivity of anti-growth signals, activating invasion and metastasis, deregulated metabolism, evading immune system, and inducing inflammation (Table S5). 

**Figure 5:**
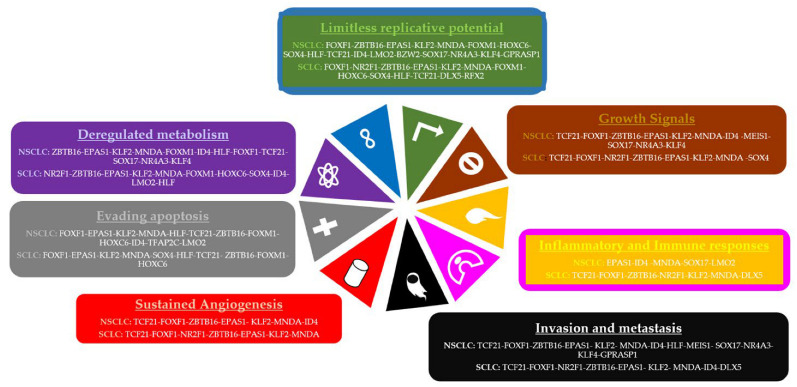
Most frequently dysregulated transcription factor networks related to the acquisition of the hallmarks of cancer [26] in non-small-cell lung cancer (NSCLC) and small-cell lung cancer (SCLC).

Eight of the most frequent TFs regulate DEGs related to angiogenesis, of which seven regulate DEGs in SCLC and PAH, and seven regulate DEGs in NSCLC and PAH. Eighteen of the most frequent TFs regulate DEGs related to limitless replicative potential, of which eleven regulate DEGs in SCLC and PAH, ten regulate DEGs in SCLC, thirteen regulate DEGs in NSCLC and PAH, and sixteen regulate DEGs in NSCLC. Fifteen of the most frequent TFs regulate DEGs related to invasion and metastasis, of which eight regulate DEGs in SCLC and PAH, eight regulate DEGs in SCLC, eight regulate DEGs in NSCLC and PAH, and twelve regulate DEGs in NSCLC. Thirteen of the most frequent TFs regulate DEGs related to growth signaling, of which seven regulate DEGs in SCLC and PAH, six regulate DEGs in SCLC, seven regulate DEGs in NSCLC and PAH, and ten regulate DEGs in NSCLC. Thirteen of the most frequent TFs regulate DEGs related to apoptosis, of which ten regulate DEGs in SCLC and PAH, and twelve regulate DEGs in NSCLC and PAH. Eleven of the most frequent TFs regulate DEGs related to inflammatory and immune responses, of which seven regulate DEGs in SCLC, two regulate DEGs in NSCLC and PAH, and four regulate DEGs in NSCLC. Sixteen of the most frequent TFs regulate DEGs related to the dysregulation of metabolism, of which eleven regulate DEGs in SCLC, nine regulate DEGs in NSCLC and PAH, and ten regulate DEGs in NSCLC (Figure 5). Furthermore, the SCLC GRNs are related to TGF-𝛽, integrin-mediated, cell surface receptor, BMP, p53, metabolic, FoxO, and cGMP-PKG signaling pathways. Meanwhile, the NSCLC GRNs are related to TGF-𝛽, integrin-mediated, adenylate cyclase-activating G-protein coupled receptor, FoxO, BMP, insulin receptor, cGMP-PKG, p53, HIF-1, AMPK, and mTOR signaling pathways (Table S5). ChEA3 analysis identified twenty-one of the most frequent TFs associated with the regulation of the most frequent LC DEGs; the other five TFs are part of their targets, related mostly by co-expression and ChIP-Seq experiments found in the literature, ARCHS4, Enrichr, ReMap, GTEx, and ENCODE databases (Table S6).

### 3.3. Coregulatory Networks and Fibration Symmetries Analysis

**Figure 6:**
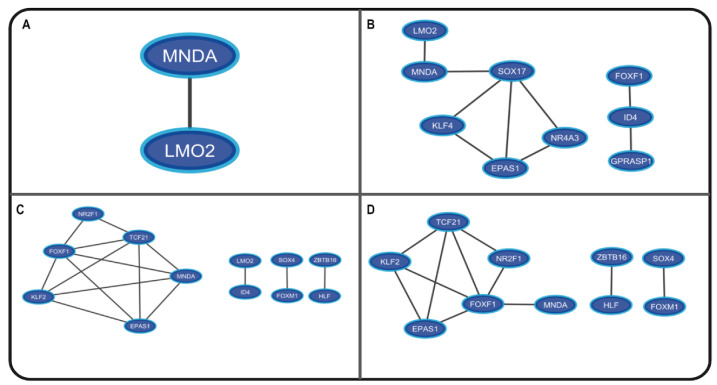
Coregulatory networks formed by the most frequently dysregulated transcription factors related to the control of gene expression of (**A**). NSCLC and PAH deregulated genes, (**B**). NSCLC deregulated genes, (**C**). SCLC and PAH deregulated genes, and (**D**). SCLC deregulated genes.

**Figure 7:**
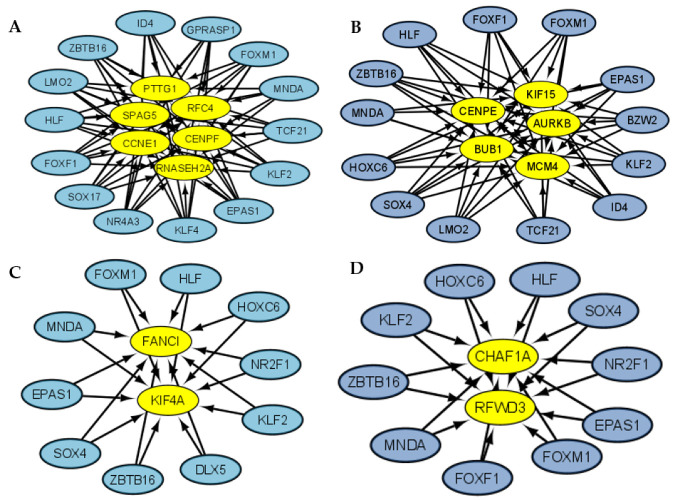
Largest blocks from fibration symmetries analysis related to the control of gene expression of (**A**) NSCLC and PAH deregulated genes, (**B**) NSCLC deregulated genes, (**C**) SCLC and PAH deregulated genes, and (**D**) SCLC deregulated genes.

The NSCLC and SCLC coregulatory networks were made based on the GRNs showing important functional associations between two, three, and six of the most frequent TFs specific to every type of lung cancer (Figure 6). The coregulatory networks or group of transcription factors might form protein–protein complexes to regulate the gene expression of DEGs unique to lung cancer in NSCLC and SCLC, and the complexes regulate common DEGs between lung cancer and PAH in NSCLC and SCLC (Table S5). The clusters of synchronized genes or fibers are the synchronized building blocks of every gene regulatory network, and symmetry fibrations are transformations that maintain information flow dynamics in the network [10]. The analysis identified thirty-five functional blocks in NSCLC and thirty-eight functional blocks in SCLC GRNs from five to fourteen regulators each (Table S7). The most frequent LC-PAH TFs form twenty-four blocks from five to thirteen regulators (Figure 7A), regulating the most frequent LC-PAH DEGs in NSCLC-GRN related to lung cancer and PAH. The most frequent LC-PAH and LC TFs form eleven blocks from nine to fourteen regulators (Figure 7B), regulating the most frequent LC DEGs in NSCLC-GRN related only to lung cancer. The most frequent LC-PAH TFs form twenty-nine blocks from two to eleven regulators (Figure 7C), regulating the most frequent LC-PAH DEGs in SCLC GRN related to lung cancer and PAH. The most frequent LC-PAH and LC TFs form nine blocks from four to ten regulators (Figure 7D), regulating the most frequent LC DEGs in SCLC-GRN related only to lung cancer.

### 3.4. Transcriptional Regulatory Network Analysis of the Most Frequently Dysregulated Transcription Factors

**Figure 8:**
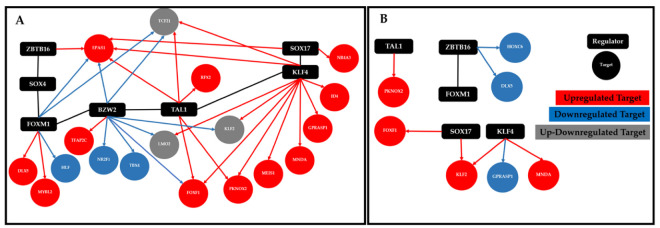
Transcriptional regulatory network of the top seven deregulated transcription factors (squares) over the other nineteen frequently dysregulated transcription factors (circles) in every lung cancer type ((**A**). NSCLC and (**A**). SCLC). In blue are the downregulated TFs, and in red are the upregulated TFs.

The RTN analysis showed the importance of the regulatory function of the top seven deregulated transcription factors (Table 2) in the regulation of the transcriptional regulatory network in NSCLC (Figure 8A) and SCLC (Figure 8B). In NSCLC, the top seven deregulated transcription factors regulate eighteen of the most frequently dysregulated transcription factors, and in SCLC, they regulate seven of the most frequently dysregulated transcription factors. 

### 3.5. Survival Analysis of the Top Seven Deregulated Transcription Factors 

**Figure 9:**
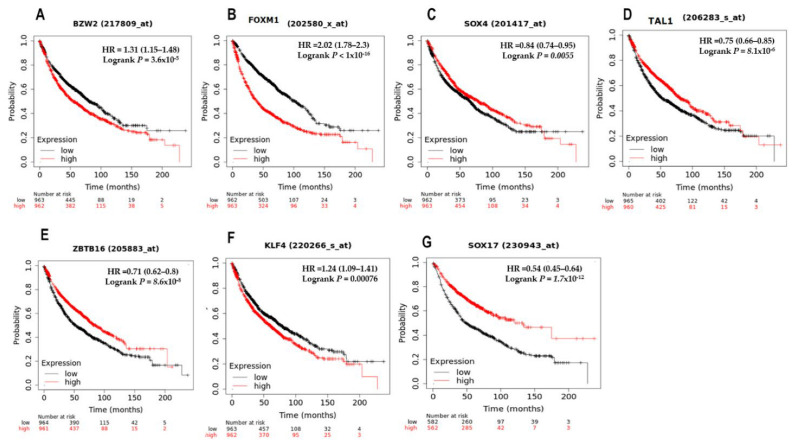
KM plots assessing the correlation between the expression of the top deregulated TFs (mRNA) and survival of lung cancer patients. (**A**) BZW2, (**B**) FOXM1, (**C**) SOX4, (**D**) TAL1, (**E**) ZBTB16, (**F**) KLF4, and (**G**) SOX17.

The Kaplan–Meier plotter analysis of the top deregulated TFs revealed a statistically significant association between the expression levels of the top five TFs deregulated also in PAH (BZW2, FOXM1, SOX4, TAL1, and ZBTB16) and the top two TFs deregulated only in lung cancer (KLF4 and SOX17), with the survival of lung cancer patients (Figure 9).

## 4. Discussion

The selected datasets represent an important number of studies that allow the gene expression of lung cancer cases and non-tumor tissue controls to be compared, in order to identify a differentially expressed gene metafirm of the disease. Datasets of the two types (NSCLC and SCLC) and subtypes (LAC and SCC) in different populations were searched to represent, as completely as possible, the dysregulation in transcriptional control processes in lung cancer. The pulmonary arterial hypertension (PAH) dataset stood out for the large number of dysregulated genes [6], much higher than lung cancer [6,12], which made it more likely to find an important number of common DEGs with lung cancer. Pulmonary arterial hypertension (PAH) is the increase in mean pressure greater than 20 mmHg, found by right heart catheterization, which is associated with the lower survival of patients with lung cancer, since it increases the rate of complications during diagnosis and the treatment [10,27]. PAH is a very complex disease similar to cancer, with an important effect of environmental stress in its etiology, such as inflammation and hypoxia, which induce the formation of hyperproliferative and apoptotic-resistant clones of different cells involved in lung tissue, which in turn influence the development of PAH-affected cells that exhibit several hallmarks of cancer [26]. Moreover, cancer and PAH shared three robust hallmarks, involved with the phenotypic, angiogenic, and glycolytic switch, along with inflammation and metabolic changes, which might be related to the cancer treatment response [28]. The most frequent DEGs found in at least eight LC datasets are related to important biological processes and signaling pathways associated with the acquisition of the hallmarks of cancer described by Hanahan and Weinberg, according to the DAVID functional annotation analysis (Table S1). Moreover, the transcriptomic analysis identified a TF regulatory network in the most frequent LC DEGs, of which some are also deregulated in PAH, and others are unique to lung cancer (Table 2). 

The common connection patterns (CCPs) formed between the most frequent LC DEGs co-expressed in LC and PAH suggest that there is an important group of genes and TFs (FOXM1 and FOXF1) that act as a group in all types and subtypes of lung cancer during the acquisition of the hallmarks of cancer, while others (TCF21, SOX17, TAL1, LMO2, KLF2, and TBX4) are important during lung cancer progression. The importance of FOXM1 and MYBL2 in regulating the biological processes that trigger lung cancer can be seen from the smallest CCP formed when comparing all co-expression networks, to the largest CCP formed among RNA-Seq studies of lung cancer (Table 3). Moreover, FOXM1 is co-expressed in the CCPs of LC microarray datasets, and PAH, FOXM1, and MYBL2 are co-expressed in the CCP of all LC microarray datasets (Table S4), suggesting their key importance during the lung cancer oncogenic process. 

**Figure 10:**
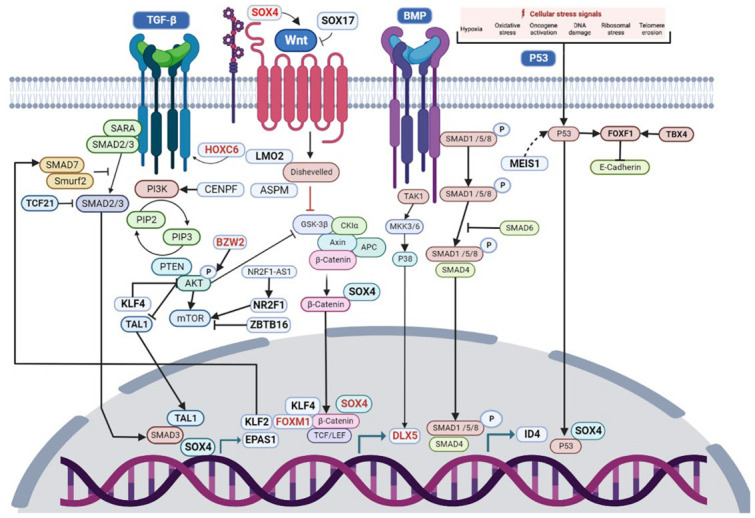
Oncogenic signaling pathways regulated by the most frequently dysregulated transcription factors in lung cancer during the acquisition of the hallmarks of cancer. Created by BioRender.

According to the DAVID analysis, FOXM1 is related to the regulation of cell growth and proliferation, while FOXM1 and MYBL2 are related to cellular senescence and the positive regulation of transcription (Table S1 and Table S4). FOXM1 has been previously identified as an essential molecular marker of NSCLC prognosis, because its expression is closely correlated with lymph node status and TNM stage, giving proliferation and invasion advantages to NSCLC cells [29]. FOXM1 increases the nuclear translocation of β-catenin and the TCF/LEF interaction (Figure 10) [30,31]. MYBL2 has been related to the proliferation and migration of NSCLC cells [32], as well as genomic instability in lung adenocarcinoma [33]. MYBL2 and FOXM1 have been identified as the upstream regulators of a local “driver network” related to NSCLC cell proliferation [34]. MYBL2 and FOXM1 were related to cancer-specific enhancers, and its high expression in lung adenocarcinomas has been related to poor patient survival [35]. The complex formed between MYBL2 and MuvB is needed to increase target specificity for FOXM1 binding. Moreover, DREAM and other MuvB-derived complexes bind to DNA through cell cycle gene homology regions (CHRs), and DREAM can associate to E2F/pRB-related components and to B-MYB and FOXM1 to regulate transcription during the cell cycle [36]. The highest expressed genes in G1 and S phases are controlled through E2F or E2F/CLE sites and can be activated by E2F1-3/DP complexes; meanwhile, genes expressed in the G2 phase and mitosis are upregulated by MMB and FOXM1-MuvB activator complexes through CHR or CDE/CHR elements [37]. 

ASPM, TPX2, TOP2A, and CENPF are the most significant Gedevo alignments (Table 4). These alignments and KIF20A appear in all datasets of CCP (Figure 3). KIF20A is upregulated in lung adenocarcinoma, and it is related to cell proliferation and apoptosis [38]. TPX2 is upregulated in NSCLC, promoting metastasis and the progression of the tumoral disease [39]. TOP2A is also upregulated in lung adenocarcinoma, and it is related to poor patient prognosis [40]. ASPM overexpression in lung adenocarcinoma has been correlated with poor prognosis [41] and tumor aggressiveness [42]. ASPM is downregulated in PAH and upregulated in lung cancer, and it has been related to the positive regulation of canonical Wnt signaling pathway (Table S1), along with a top deregulated LC-PAH TF SOX4, and a top deregulated LC TF DLX5, suggesting that it might be related to the regulation of this pathway in lung cancer (Figure 10). ASPM regulates the expression of N-cadherin, vimentin, and Snail during epithelial–mesenchymal transition, promoting cell invasion in NSCLC cells and the activation of Wnt/β-catenin signaling pathway (Figure 10) [43]. The ASPM/Dishevelled signal axis is highly activated in superpotent CSCs (Figure 10) [44]. 

**Figure 11:**
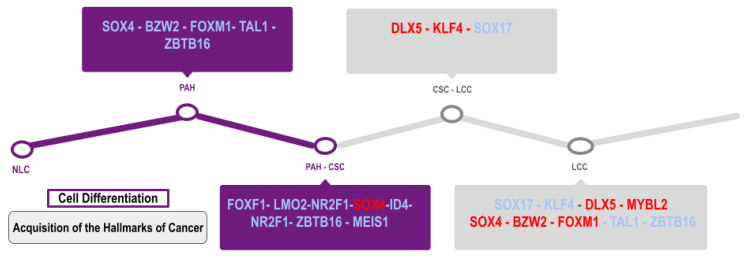
Model of the acquisition of cancer stem characteristics and lung cancer cell differentiation regulated by the most frequently dysregulated transcription factors, simultaneous to the acquisition of the hallmarks of cancer. First are normal lung cells (NLC), then pulmonary arterial hypertension cells (PAH), cancer stem cells (CSC), and differentiated lung cancer cells (LCC).

CENPF (Centromere protein F) is a gene related to chromosome segregation during mitosis that reduces overall survival in lung cancer and is a hub gene (AURKB, BUB1B, KIF2C, HMMR, CENPF, and CENPU) overexpressed in cancer patients [45]. CENPF is one of the most frequently dysregulated LC DEGs related to cell division and differentiation, along with seven of the most frequently dysregulated LC-PAH TFs (FOXF1, LMO2, SOX4, ID4, NR2F1, ZBTB16, and MEIS1) (Table S1), and three of the most frequently dysregulated lung cancer TFs (SOX17, DLX5, and KLF4), suggesting a role in the process of cancer stem cells’ differentiation into lung cancer cells (Figure 11). CENPF expression has been related to lung adenocarcinoma progression through the regulation of the ERβ2/5 signaling pathway [46] and the PI3K–AKT–mTORC1 signaling pathway (Figure 10) [47]. CENPF overexpression is positively associated with an advanced differentiation stage and a shorter overall survival, making it a risk factor for the cancer prognosis related to the ability of tumor cell proliferation and migration [48,49]. CENPF is one of the overexpressed genes that appeared in three of the LC CCPs and formed five different alignments with Gedevo, one of which was in 60% of the possible comparisons (Table 3), suggesting its importance in lung cancer. The overexpression of FOXM1 and CENPF in prostate cancer have been linked to the loss of microRNAs such as miR-101 and miR-27a, to the synergistic cancer induction through the upregulation of PI3K and MAPK signaling pathways, and to the poor prognosis prediction of cancer patients [50]. Combining functional pathways and protein–protein interaction analyses, five hub genes (CDC20, CENPF, KIF2C, BUB1, and ZWINT) were identified, which are also in the CCPs, and two of which are in the Gedevo analysis [51]. 

FOXF1 (forkhead box F1) is a stemness reprogramming mediator (when mesenchymal stem cells fuse with lung cancer cells) which is also related to the inhibition of cell growth, proliferation, and migration [52]; therefore, it must be downregulated in lung cancer cells (Table 1). FOXF1 downregulation in lung-resident mesenchymal stromal cells is associated with upregulation of genes important for the regulation of high cell proliferation, migration, and inflammatory responses [53]. FOXF1 is co-expressed in the CCP of LC RNA-Seq and PAH, suggesting that it is an important regulator for the tumoral process (Table S5). FOXF1 is related to the positive regulation of cell migration, transcription, and the negative regulation of inflammatory response (Table S1), suggesting its importance as a tumor suppressor gene regulating the inflammation, invasion, and metastasis of cancer stem cells (CSC) (Figure 11). In vitro hypomethylation of FOXF1 is able to increase its expression, thus inhibiting cell apoptosis induced by cisplatin, promoting cell proliferation and the expression of stem cell characteristics and self-renewal ability, which also suggests that it could be a prognostic biomarker of platinum-based chemotherapy resistance in NSCLC [54]. FOXF1 overexpression inhibits vascular endothelial growth factor A1 (VEGFA) in in vitro and in vivo attenuated angiogenesis [55]. FOXF1 expression is upregulated by p53, tAp63, and tAp73, which directly binds to its promoter and decreases E-cadherin expression, inhibiting cell invasion and migration (Figure 10) [56]. FOXF1 is upregulated in lung cancer CAFs by the hedgehog signaling pathway, which might be related to their ability to modulate the inflammatory response and stimulate tumor cell growth, invasion, angiogenesis, and metastasis [57]. Moreover, FOXF1 is also co-expressed in the CCP of the LC RNA-Seq datasets, along with seven of the most frequently dysregulated TFs (FOXM1, SOX17, TAL1, TCF21, TBX4, LMO2, and KLF2), suggesting its importance as a regulator of gene expression during lung cancer progression. 

TCF21 (transcription factor 21) is a tumor suppressor gene that is methylated and downregulated in lung cancer and is related to cell viability, proliferation [58], apoptosis, and growth [59], as well as angiogenesis, epithelial–mesenchymal transition, tissue invasion, and metastasis [60]. Moreover, TCF21 overexpression is related to chromatin accessibility blocking at the SMAD3 binding site, inhibiting the SMAD3 function of gene expression regulation (Figure 10). TBX4 (T-box 4) regulates lung branching morphogenesis and vascular development, maintaining proper tissue homeostasis during lung development through the interaction of TBX4-FGF10 and SHH-FOXF1 cascades [61]. TBX4 is a tumor suppressor in lung adenocarcinoma and NSCLC [62] whose expression is regulated by a methylating pattern, avoiding its inhibition of cell growth and proliferation or the induction of apoptosis [63]. TAL1 (T-cell acute lymphocytic leukemia 1) is a basic helix–loop–helix (bHLH) TF that is important for hematopoietic commitment and physiological and pathological vascular processes [64]. TAL1 promotes the expression of the kinase insert domain receptor to activate the TGF-β signaling pathway [65]. TAL1 interacts with SMAD3 and strengthens the positive or negative regulation of SMAD3, including TGF-β1 inhibition (Figure 10) [66]. SCL/TAL1 interrupting locus (STIL) promotes proliferation, invasion, and cancer progression by regulating the expression of CDK1, CCNB2, CDC20, CCNA2, BUB1, and AURKB [67]. 

LMO2 (LIM-domain only 2) is an important regulator of embryonic hematopoiesis [68] and angiogenesis [69]. The downregulation of LMO2 occurs due to the establishment of tumorigenesis, inhibiting apoptosis and promoting cell proliferation, migration, invasion [70], and tumor growth through the Wnt signaling pathway [71]. LMO2 has been correlated to oncogenic pathways related to the regulation of stemness and epithelial–mesenchymal transition, PPAR, TGF-beta/BMP, and mTOR pathways, central carbon metabolism, cell senescence [72], and genomic instability [73]. KLF2 (Kruppel-like factor 2) is a tumor suppressor whose downregulation is related to region 4 hypermethylation in NSCLC tissues, and it is associated with lymph node metastasis and advanced TNM stage, cell viability, cell cycle, inhibition of apoptosis [74], promotion of cell growth, cell survival and proliferation [75], and angiogenesis, improving vascular stability [76], vascular permeability in NSCLC [77], and inflammation, possibly through the regulation of AP-1 [78]. KLF2 expression is related to the inhibition of TGF-β signaling through the induction of Smad7 (Figure 10) [79]. KLF2 upregulation decreases the glutamine level and participates in the consumption of glutamine by NSCLC cells, inhibiting its energy metabolism [80].

SOX17 belongs to sex-determining region Y (Sry), a box-containing family of transcriptional regulators that are essential for stem cell maintenance, lung morphogenesis, and tissue homeostasis [81]. SOX17 in mice tumor endothelial cells promotes tumor progression, angiogenesis, and vascular destabilization [82]. Notch intracellular domain overexpression downregulates SOX17 expression in primary endothelial cells, avoiding the excessive tip cell formation and hyperbranching of the vascular network during development and tumor angiogenesis [83]. The downregulation of SOX17 might also be related to the promoter methylation of CpG sites, suggesting that demethylating drugs would be a promising approach for lung cancer treatment [84]. SOX17 regulates respiratory epithelial cell differentiation [85]; therefore, it must be downregulated (Table 1), or its upregulation could avoid epithelial–mesenchymal transition in lung cancer cells, probably as a mutated cancer driver gene and a re-engineered reprogramming factor through the cross-talk with the WNT/β-catenin pathway (Figure 10) [86]. SOX17 overexpression acts as a tumor suppressor of cancer cell growth, proliferation, migration, and invasion [87]. 

The GRN analysis of lung cancer types associated the hallmarks of cancer with the target DEGs of twenty-three of the most frequently dysregulated TFs (Figure 5). Eight out of the nine deregulated TFs that are important in the co-expression network analysis are also important for the regulation of the most frequent LC DEGs (FOXM1, FOXF1, TCF21, TBX4, TAL1, LMO2, KLF2, and SOX17), along with sixteen other deregulated TFs (BZW2, DLX5, SOX4, ID4, NR2F1, EPAS1, ZBTB16, MNDA, HOXC6, HLF, RFX2, NR4A3, KLF4, GPRASP1, MEIS1, and TFAP2C). However, MYBL2 is only co-expressed in lung cancer microarray CCP, TBX4 is only co-expressed in lung cancer RNA-Seq CCP, PKNOX2 is not co-expressed in any CCP, and neither is a regulator in the GRNs. BZW2 (basic leucine zipper and W2 domains 2) overexpression in lung adenocarcinoma has been related to tumor size, stage, and lymphatic invasion [88]. BZW2 knockdown inhibits cell proliferation and promotes cell apoptosis within the LINC00174/miR-4500/BZW2 axis, possibly through the inactivation of the AKT/mTOR signaling pathway [89]. Therefore, BZW2 overexpression suggests that it is an oncogene in lung cancer related to the phosphorylation of the components of the AKT/mTOR pathway (Figure 10).

DLX5 (distal-less homeobox 5) is mainly related to embryonic and postnatal development and cell differentiation, and it is overexpressed in lymphomas and lung cancer [90]. DLX5 is methylated in early stage lung cancers [91], suggesting that it is only deregulated in lung cancer progression, and it is not in PAH (Table 1), in which it induces the expression of MYC and β-catenin, promoting cell proliferation and metastasis (Figure 10) [92]. The downregulation of miR-339-5p expression also increases DLX5 expression, inducing stem cell differentiation and the activation of the Wnt/β-catenin signaling pathway [93], which could suggest its participation in the differentiation process of CSC in lung tumor cells (Figure 11). SOX4 belongs to the SRY-related HMG box family of TFs; it is related to embryonic development and cell-fate differentiation [94] and promotes epithelial–mesenchymal transition and stemness of cancer cells, and TGF-β is related to the upregulation of its expression in cancer cells (Figure 10) [95]. SOX4 is related to the positive regulation of transcription, cell differentiation and proliferation, and the positive regulation of the canonical Wnt signaling pathway (Table S1). SOX4 overexpression in lung cancer is related to the mechanisms of gene amplification, and the active form synergizes to promote cell growth along with the RHOA-Q63L oncogene, suggesting its importance as a lung cancer oncogene [96]. 

SOX17 represses and SOX4-enhanced canonical Wnt signaling (Figure 10) to keep up CSC proliferation, self-renewal, and differentiation, thus assisting the invasion and metastasis of lung cancer through the regulation of Wnt target genes mainly related to cell cycle, stem cell pluripotency, and epithelial–mesenchymal transition (Figure 11) [81]. Therefore, SOX17 must be downregulated and SOX4 upregulated (Table 1) to allow for the establishment and progression of lung cancer. The p53/miR-30a-5p/SOX4 feedback loop has been related to NSCLC cell proliferation, apoptosis, and invasion [97]. The HMG box domain of SOX4 interacts with p53, repressing p53-mediated apoptosis (Figure 10) [98]. SOX4 regulates melanoma glycolytic metabolism controlling the transcriptional expression of glucose transporter type 1, hexokinase 2, and lactate dehydrogenase A, and activates mTORC1 to promote proliferative signals [99] and cell growth when SOX4 is upregulated by CD147 [100]. 

ID4 belongs to the DNA-binding (ID) protein family, which are dominant negative inhibitors of basic helix–loop–helix (bHLH) transcription factors, regulating developmental processes and promoting stem cell survival, differentiation, and epigenetic inactivation of gene expression in late cancer stages [101]. ID4 is related to cell differentiation and the positive regulation of transcription and gene expression (Table S1). ID4 is a reprogramming factor that differentiates glioma cells and immortalized astrocytes to glioma CSCs [102]. The activation of the PDGF-NO-ID4 axis promotes tumor progression, increasing CSC self-renewal and tumor angiogenesis [103]. ID4 regulates factors associated with angiogenesis [104], among which there are regulators of inflammatory responses such as AOC3, AGTR1, CDO1, PTGER4, SPP1, SELP, and SDC1 (Table S1). ID4 is a tumor metastasis suppressor regulating EMT in lung adenocarcinoma [105], increasing cell apoptosis, and inhibiting cell proliferation through S-phase progression arrests [101]. ID4 may inhibit colorectal cancer cell growth, epithelial–mesenchymal transition, and metastasis, thus inhibiting the PI3K/AKT pathway [106]. The BMP-Smad1-Id pathway regulates the acquisition of the oncogenic phenotype in Kaposi’s sarcoma (Figure 10) [107]. ID4 is involved in cell metabolism and transcription regulation in the pathogenesis of lung cancer and could become a biomarker of lung cancer occurrence and prognosis [108]. 

NR2F1 or Coup-TF1 (nuclear receptor subfamily 2, group F, member 1) is an orphan nuclear receptor of the retinoic acid receptor family, known as a tumor dormancy marker, which is downregulated in cancer to promote cell proliferation [109] and metastasis by inducing the epithelial–mesenchymal transition [110,111]. NR2F1 downregulation is related to high cancer mutation rates, immune responses, and cell infiltrations, and it is upregulated in inflammatory cancer-associated fibroblasts (CAFs) [109]. NR2F1 downregulation might be related to the activation of cell growth and the inhibition of apoptosis during cell differentiation [112]. Long noncoding RNA NR2F1-AS1 is upregulated by the hypoxia inducible factor, promoting cell proliferation, migration, and invasion through the activation of the NR2F1/AKT/mTOR pathway (Figure 10) [113]. NR2F1-AS1 induces NSCLC cell tumorigenesis sponging miR-363-3p in order to increase SOX4 [114]. Hypoxia in the tumor microenvironment promotes excessive angiogenesis, metabolic reprogramming, immune escape, cell proliferation, and metastasis [115]. NR2F1-AS1 is upregulated under hypoxia, triggering hypoxia-related glycolysis and migration through the miR-140/HK2 pathway [116]. 

EPAS1 (endothelial PAS domain-containing protein 1 or hypoxia-inducible factor 2 alpha (HIF2α)) is related to vascular network remodeling [117], tumor angiogenesis, tumor size, tissue invasion [118], metastasis, cell dedifferentiation, enhanced glycolytic metabolism, antiapoptotic activity, and genomic instability [119]. EPAS1 is upregulated in PAH and downregulated in eight datasets of lung cancer (Table 1), suggesting that it could be an important oncogene during the lung cancer tumorigenic process. Hypoxic-stabilized EPAS1 proteins transactivate DNMT1, promoting EPAS1 promoter hypermethylation and thus decreasing EPAS1 mRNA levels, which is much lower in poorly differentiated tumors compared with well and moderately differentiated ones, indicating that it can be a poor prognosis marker of NSCLC [120]. EPAS1 rs4953354 polymorphism is related to gene expression and NSCLC susceptibility, specifically in female never- smokers with lung adenocarcinoma [121], along with DNA methylation regulation of mRNA levels [122]. Hypoxia-inducible factors represent an adaptive mechanism to promote tumor growth under hypoxic microenvironments through direct cytokine and ROS production as well as angiogenesis, a signaling switch for pro-tumorigenic inflammatory responses through the recruitment of pro-tumor immune cells, and an effector that suppresses antitumor immune responses [123].

ZBTB16 (zinc finger and BTB domain-containing 16, promyelocytic leukemia zinc finger protein (PLZF), or zinc finger protein 145 (ZFP145)) is a tumor suppressor gene that is downregulated by promoter hypermethylation, which stimulates cancer cell proliferation, migration, invasion [124], metastasis [125], high tumor grade, tumor stage, and shorter overall survival in NSCLC [126]. ZBTB16 balances stem cell differentiation and self-renewal in a cell-type-specific manner [127], as well as cell growth [128], differentiation, and apoptosis [129,130]. ZBTB16 is an intrinsic factor that suppresses mTORC1 activity in stem cells to maintain self-renewal capacity (Figure 10) [131]. ZBTB16 targets LC DEGs that are related to the induction of angiogenesis (Table S5) [132]. ZBTB16 is involved in almost all processes underlying the pathogenesis of metabolic syndrome, mainly related to immune function, inflammation, and oxidative stress [133]. ELFN1-AS1 recruits DNMTs to the ZBTB16 promoter and silences its expression, leading to the activation of the PI3K/AKT signaling pathway and tumorigenesis [134] and the inhibition of the MAPK pathway [135]. ZBTB16 downregulation is related to a higher expression of inflammatory cytokines and initiates an amplified inflammatory response to infectious stimuli [136].

MNDA (myeloid cell nuclear differentiation antigen) is related to innate immunity [137]. Its downregulation is controlled by transcriptional and posttranscriptional mechanisms such as methylation and miRNAs (hsa-miR-33a-5p and hsa-miR-33b-5p) in lung adenocarcinoma. It is related to immune cell infiltration [138], the increase of cell proliferation, migration, invasion, growth, as well as the inhibition of apoptosis [139]. MNDA binds directly to YY1, enhancing YY1 affinity for its target DNA, keeping the association stable longer, and giving lineage-specific features to the YY1 function [140]. YY1 is overexpressed in NSCLC and co-expressed in the NSCLC gene network [6], probably related with the activation of cell proliferation and invasion [141], forming a regulatory loop with cancer stem cell transcription factors (SOX2, OCT4, and BMI1) in the NF-kB/PI3K /AKT axis [142]. 

HOXC6 (homeobox C6) is overexpressed in lung cancer, regulating the expression of genes related to cell proliferation, migration, and invasion in NSCLC [143]. HOXC6 regulates EMT, high immune cells infiltration, the expression of immune checkpoint genes [144], cell migration, invasion [145], cell growth [146], cell proliferation [147], cell apoptosis, and viability through the TGF-β/smad signaling pathway (Figure 10) [148]. HLF (hepatic leukemia factor) downregulation is related to genetic deletions and methylation, to distant NSCLC cells metastasis, promoting anaerobic metabolism to support NSCLC cell growth in a low nutritional environment [149]. HLF has an anti-apoptotic program characterized by the upregulation of specifically related genes, and the downregulation of pro-apoptotic genes [150]. RFX2 (DNA-binding protein RFX2) dysregulation is characteristic of SCLC, which could become a diagnostic marker, key for the development of molecular-targeted drugs [151]. NR4A3 (Nuclear receptor subfamily 4 group A member 3) is a tumor-suppressive gene with p53-dependent and -independent functions, which is a direct transcriptional target of p53 and related to cell proliferation, migration, and apoptosis [152], and elevating the intracellular levels of reactive oxygen species [153]. NR4A3 regulates genes involved in inflammatory response, complement activation, metabolism [154], pro-inflammatory signaling, cell proliferation, growth, apoptosis, survival, migration, angiogenesis, and tumor immune surveillance [155]. 

KLF4 (Krüppel-like factor 4) induces pluripotent stem cells, controls cell fate reprogramming and self-renewal of embryonic stem cells (Figure 11) [156]. KLF4 acts as a negative regulator of the AKT/GSK3β pathway during cell differentiation (Figure 10) [157]. KLF4 is a tumor suppressor, and its downregulation is related to class I histone deacetylases, lung inflammation in conjunction with K-ras activation, tumorigenesis, the modulation of cell proliferation [158], cell growth [159], epithelial–mesenchymal transition [160], invasion, and metastases [161]. KLF4 downregulation activates hTERT and telomerase activity, MAPK signaling, and thus lung cancer cell growth [161]. Cancer cells sustain growth under metabolic stress due to the Warburg effect, so KLF4 downregulation is involved in metabolic pathways that respond to low glucose, increased reactive oxygen species (ROS), and decreased autophagic response to glucose starvation. Therefore, KLF4 has a non-Warburg metabolic behavior as a tumor suppressor gene [162]. GPRASP1 (G protein coupled receptor-associated sorting protein 1) is deregulated in several types of cancer [12], and its downregulation is related to the inhibition of the Tachykinin Receptor family, which is involved in inflammation and cancer cell proliferation [163]. 

MEIS1 (Meis homeobox 1) downregulation is related to lung adenocarcinoma cell proliferation [164], anchorage-independent growth, cell cycle progress, apoptosis, invasion, and migration [165]. The suppression of MEIS1 expression is related with epigenetic regulation mediated by EZH2-DNMT3a and lncRNA ELFN1-AS1, cell viability and tumor growth [166], and CpG island methylation of squamous cell carcinomas and lung adenocarcinomas cells [167]. MEIS1 inhibition regulates angiogenesis [168], cell growth [169], expression of stem cell markers [170], self-renewal, proliferation, differentiation of human pluripotent stem cells (hPSCs), the upregulation of cell cycle regulators such as checkpoint kinase 1 (CHEK1) and cyclin D2 (CCND2), and the downregulation of negative regulators of the cell cycle such as tumor protein p53 (Figure 10) [171]. MEIS1 inhibition downregulates Hif-1α and Hif-2α in hematopoietic stem cells (HSCs), causing a shift to mitochondrial metabolism, increasing reactive oxygen species production and maintaining HSCs [172]. MEIS family genes can promote or inhibit cancer probably through the different degrees of immune silencing [173] and the significant correlation of MEIS1 with immune genes according to a gene regulatory network analysis [174]. Proteoglycans in cancer is the most enriched pathway associated with MEIS1 and HOXB13 inhibition, inducing tumor suppression and DCN, LUM, and TGFBR3, as well as regulating growth factor, migration, and invasion signaling through receptor tyrosine kinases [175]. TFAP2C (transcription factor activating enhancer-binding protein 2C) overexpression in NSCLC is related to cell proliferation and the downregulation of GADD45B and PMAIP1 [176]. TFAP2C upregulation induces cell cycle hyperactivation, disease aggressiveness through the miR-183 and miR-33a pathways [177], cell cycle progression, cell viability, proliferation, motility, and migration [178]. 

The transcriptional regulatory network of most frequently dysregulated TFs showed consistent evidence of its ability to accomplish their regulatory function throughout the course of lung cancer, in the CCP, regulatory, coregulatory, and fibration symmetries analysis, by means of a cooperative and coordinated function during the acquisition of each hallmark of cancer (Figure 5), each biological process (Figure 7), and each signaling pathway (Figure 10), as well as forming co-regulatory complexes to control transcriptional expression in a specific way (Figure 6). Likewise, the top deregulated TFs may form a transcriptional regulatory network (Figure 8) to regulate the transcriptional expression of the other frequently deregulated TFs and therefore be at the top of the gene regulation inside oncogenic signaling pathways related to the acquisition of the hallmarks of cancer (Figure 10). 

In NSCLC, for RTN, there seems to be a regulatory function of the top deregulated TFs over the other TFs (Figure 8A), suggesting the importance of the top seven TFs regulating the oncogenic processes. NSCLC regulatory complexes might be formed by other types of cofactors or proteins because the coregulatory networks are small, both in TFs with targets in lung cancer and PAH (Figure 6A) and targets unique to lung cancer (Figure 6B), so the regulatory function is accomplished with the coordinated activity of the most frequently dysregulated TFs as verified by the number of regulators in the blocks related to NSCLC and PAH (Figure 7A) and only to NSCLC (Figure 7B). However, in SCLC, the RTN does not seem to have this regulatory function of the top TFs over the other frequently deregulated TFs (Figure 8B), but the regulatory complexes that are formed to regulate targets in SCLC and PAH (Figure 6C) and only in SCLC (Figure 6D) are larger, suggesting that these are the ones in charge to fulfill the regulatory function in a cooperative manner. This is also justified by the number of regulators in the blocks that might be related to SCLC and PAH (Figure 7C) and only to SCLC (Figure 7D). The regulatory function of the lung cancer transcriptional regulatory network of the most frequently dysregulated TFs over the most frequent LC DEGs has been experimentally validated by multiple ChIP-Seq and co-expression independent studies in several tissues [23]. Moreover, most of the transcriptional regulatory network is also important for other types of cancer, with FOXF1, HOXC6, and RFX2 being the only ones that seem to be lung-cancer-specific biomarkers, and even though SOX4 and SOX17 are deregulated in all lung cancer datasets, they are also deregulated in some types of breast cancer and leukemia, suggesting a more general tumor function [12]. Consequently, it is the coordinated and cooperative regulatory function of the transcriptional network of transcription factors that may be related to the acquisition of the hallmarks of cancer during the tumor process and each type and subtype of lung cancer. 

The Kaplan–Meier plotter analysis of the top deregulated TFs revealed a strong association of their expression with a decreased probability of long-term survival (Figure 9). The KM plot analysis showed that the overregulation of three oncogenes (SOX4, BZW2, and FOXM1) as well the downregulation of four tumor suppressors (SOX17, ZBTB16, TAL1, and KLF4) is associated with worse overall survival (OS) for lung cancer patients. The statistically significant association between the expression levels of the TFs with a poor prognosis of lung cancer patients suggests that these TFs are important for the oncogenic disease and could become new targets for the diagnosis, treatment, and prognosis of lung cancer. Controlling the expression of a specific group of oncogenic and tumor-suppressive transcription factors might lead to selective death of cancer cells, because healthy cells are able to tolerate the loss of TF function with slight consequences, due to the presence of proteins in the transcriptional regulatory network that are capable of supplying the missing function in normal signaling pathways [179].

In the last decade, new strategies have been developed for targeting oncogenic and rescuing tumor-suppressive TF functions, modulating their expression or degradation by blocking protein/protein interactions, and preventing its DNA binding either through a binding pocket or at the DNA-interacting site; some of these inhibitors are currently being used or evaluated for cancer treatment [180]. Cortezomib or Velcade is a compound that can directly degrade a TF using the ubiquitin-proteasome or sumoylation processes [181]. Another strategy is associated with the inhibition of a TF activity blocking its DNA binding using synthetic oligodeoxynucleotide decoys, which are double-stranded nucleotide sequences derived from conserved genomic regulatory elements that are recognized by the selected TF, avoiding its ability to bind with other proteins [179]. The pharmacological inhibition of FOXM1 expression at a transcriptional, translational, and post-translational level and/or its interactions with target sites, blocking DBD, nuclear localization, or protein–protein interaction may be an effective way to inhibit FOXM1 oncogenic mechanisms of action [182]. Thiazolidinedione (TZD) inhibits FOXM1 expression through the downregulation of SP1, negatively regulating tumor cell growth and promoting apoptosis [183]. Diarylheptanoids can also suppress FOXM1 expression, suppressing Gli1 in pancreatic cancer cells [184]. The regulation of TFs to treat cancer is a current research field that is continually improving and able to develop specific and more effective strategies to control the abnormal gene expression patterns of cancer. 

## 5. Conclusions

Our bioinformatic pipeline allowed for the identification and analysis of the positively and negatively dysregulated genes in most of the lung cancer datasets, which are dysregulated only in lung cancer, or dysregulated also in PAH, to identify an essential group of co-expressed TFs, which might be related to the overall tumoral process, independently of the type or subtype of lung cancer. There is experimental evidence that supports that the transcriptional regulatory network of TFs identified perform an important number of key functions during the acquisition of the hallmarks of cancer, through the regulation of the gene expression of oncogenes and tumor suppressors associated specifically with the tumoral process of the lung. Two of the most frequently dysregulated transcription factors are co-expressed in lung cancer and PAH (FOXM1 and FOXF1), while the other six frequently deregulated transcription factors are co-expressed only in lung cancer (TCF21, SOX17, TAL1, LMO2, KLF2, and TBX4). Moreover, twenty-four of the most frequently dysregulated transcription factors regulate the most frequent DEGs, from which eight are also in the co-expression analysis. The coregulatory analysis identified sixteen of the most frequently dysregulated transcription factors capable of forming protein–protein complexes to regulate gene transcription in a cooperative manner. The fibration symmetries analysis identified groups of genes that regulated up to fourteen of the frequently deregulated transcription factors, which might regulate gene expression in a cooperative and/or coordinated manner. 

The seven top deregulated transcription factors (SOX4, SOX17, BZW2, FOXM1, ZBTB16, TAL1, and KLF4) consistently appear to be co-expressed with other frequent DEGs and transcription factors throughout the analysis in lung cancer and PAH. Their regulatory function might be related to the formation of co-regulatory complexes and biological functional blocks or fibers, which seem to be able to regulate the function of the other frequent transcription factors, at least in NSCLC, and are associated with the survival of lung cancer patients. The functional analysis and experimental evidence show an association between the frequently deregulated transcription factors with the control of signaling pathways related to the acquisition of the hallmarks of cancer. Moreover, there is evidence of the functional association between the hub TFs, as cooperators or coregulators, supporting a strong transcriptional regulatory network connected with lung cancer. 

The analysis of the overall transcriptomic changes in the lung cancer oncogenic process, which was performed through the creation of co-expression, regulatory, coregulatory, and transcriptional networks of the most frequently dysregulated DEGs in lung cancer, allowed for the identification of potential biomarkers for lung cancer diagnosis for the future development of specific and more efficient anticancer therapies. The coordinated and cooperative biological function of the transcriptional regulatory network must be evaluated experimentally to fully understand and validate their importance in the regulation of signaling pathways related to the acquisition of the hallmarks of cancer, and to use them as specific biomarkers for the diagnosis and treatment of complex tumor diseases in the lung.

## Figures and Tables

**Table 1:** Gene expression datasets, each study code, and number of samples.

Study Code	Samples
GSE19804	Normal (60) vs. Cancer NSCLC (60)
GSE10072	Normal (49) vs. Cancer-Lung adenocarcinoma (58)
GSE3268	Normal (5) vs. Cancer-Squamous lung cancer cells (5)
GSE108055	Normal (9) vs. Cancer-Small-cell lung cancer (54)
E-MTAB-5231	Normal (18) vs. Cancer-NSCLC (22)
E-MTAB-3950	Normal (30) vs. Cancer-Early Squamous Carcinoma (30)
GSE52248	Normal (6) vs. Lung adenocarcinoma (12)
GSE70089	Normal (3) vs. Lung carcinoma (3)
GSE81089	Normal (19) vs. Cancer NSCLC (199)
GSE84776	Normal (9) vs. Squamous lung cancer (9)
GSE113439	Normal (11) vs. Pulmonary arterial hypertension (PAH) (15)

**Table 2:** The most frequently dysregulated lung cancer transcription factors and their fold change. In blue are the downregulated transcription factors, and in red the upregulated transcription factors in lung cancer and pulmonary arterial hypertension (PAH). The gray shading highlights the transcription factors that are dysregulated only in lung cancer.

	Transcription Factor	Lung Cancer	Fold Change	Pulmonary Arterial Hypertension	Total
1	SOX4	10	2.04040127	PAH	11
2	SOX17	10	0.50804133	-	10
3	BZW2	9	1.86844644	PAH	10
4	FOXM1	9	2.92595918	PAH	10
5	ZBTB16	9	0.36129524	PAH	10
6	TAL1	9	0.71569014	PAH	10
7	KLF4	9	0.34963135	-	9
8	EPAS1	8	0.29798652	PAH	9
9	HOXC6	8	1.64583097	PAH	9
10	ID4	8	0.58410489	PAH	9
11	KLF2	8	0.35253951	PAH	9
12	MEIS1	8	0.53393586	PAH	9
13	NR2F1	8	0.65817465	PAH	9
14	TBX4	8	0.66704351	PAH	9
15	TCF21	8	0.25969303	PAH	9
16	TFAP2C	8	1.86076131	PAH	9
17	LMO2	8	0.43204536	PAH	9
18	MNDA	8	0.43069329	PAH	9
19	FOXF1	8	0.25699375	PAH	9
20	HLF	8	0.51723087	PAH	9
21	RFX2	8	0.80986159	PAH	9
22	DLX5	8	1.76223624	−	8
23	MYBL2	8	1.79966863	−	8
24	NR4A3	8	0.39647141	−	8
25	PKNOX2	8	0.64308105	−	8
26	GPRASP1	8	0.77475237	−	8

**Table 3:** Common connection patterns (CCPs) of lung cancer (LC) (microarrays (MA) and RNA-Seq (RNAS)) and pulmonary arterial hypertension (PAH), iRegulon main regulators (FOXM1 and MYBL2), number of targets, number of binding motifs (BM), and normalized enrichment score of the motifs (NES).

CCPs	CCPs		FOXM1			MYBL2		
	Nodes	Edges	Targets	BM	NES	Targets	BM	NES
ALL LC—PAH	9	11	9	6	10.190	8	1	5.598
MA LC—PAH	39	91	30	6	10.318	19	1	6.552
RNAS LC—PAH	32	36	24	6	11.294	13	1	4.127
ALL LC	29	39	21	6	10.427	19	1	6.352
MA LC	94	555	47	6	9.595	40	1	5.509
RNAS LC	118	370	53	6	11.199	26	1	4.395

**Table 4:** Gedevo most common alignments in lung cancer co-expression networks.

Alignment	Number of Alignments	Percentage	Median
ASPM—ASPM	6	60%	0.512949636
CENPF—CENPF	6	60%	0.525433165
PRC1—PRC1	6	60%	0.446116868
TPX2—TPX2	6	60%	0.570271269
TOP2A—TOP2A	5	50%	0.551322899
KIF20A—KIF20A	4	40%	0.444933142
KIF2C—KIF2C	4	40%	0.469979467
NUSAP1—NUSAP1	4	40%	0.484566649

**Table 5:** Gene regulatory networks (GRNs) of the most frequently dysregulated transcription factors (TFs) of the two types of lung cancer: non-small-cell lung cancer (NSCLC) and small-cell lung cancer (SCLC). The number of most frequently dysregulated DEG targets in lung cancer and pulmonary arterial hypertension (LC-PAH) and only in lung cancer (LC) of each most frequently dysregulated transcription factor (TF).

	NSCLC				SCLC			
	LC-PAH		LC		LC-PAH		LC	
	TF	Targets	TF	Targets	TF	Targets	TF	Targets
1	TCF21	176	TCF21	76	MNDA	127	DLX5	67
2	ZBTB16	175	ZBTB16	74	ZBTB16	123	MNDA	66
3	FOXF1	173	FOXF1	73	KLF2	120	KLF2	64
4	FOXM1	172	NR4A3	73	EPAS1	113	EPAS1	62
5	EPAS1	164	KLF2	73	SOX4	90	ZBTB16	55
6	KLF2	164	KLF4	73	NR2F1	90	NR2F1	50
7	ID4	151	EPAS1	71	FOXF1	87	FOXF1	39
8	MNDA	143	ID4	67	HLF	63	SOX4	36
9	HLF	82	SOX17	67	HOXC6	57	TCF21	35
10	LMO2	52	GPRASP1	51	FOXM1	47	HLF	26
11	HOXC6	47	MNDA	50	TCF21	45	HOXC6	20
12	SOX4	24	HLF	31	LMO2	37	FOXM1	18
13	TFAP2C	12	FOXM1	23	ID4	34	LMO2	10
14	BZW2	10	LMO2	21	RFX2	8	RFX2	9
15	MEIS1	10	HOXC6	12			ID4	8
16	TAL1	3	MEIS1	10				
17			SOX4	6				
18			TFAP2C	3				
19			TAL1	1				
20			PKNOX2	1				
21			RFX2	1				

## Data Availability

All microarray datasets are fully available in the Gene Expression Omnibus (GEO).

## Supplementary Materials

The following supporting information can be downloaded at: https://www.mdpi.com/article/10.3390/cimb45010029/s1, Table S1. List of lung cancer (LC) and pulmonary arterial hypertension (PAH) DEGs. Lists of the most frequent LC downregulated and overregulated DEGs. Lists of the most frequent LC DEGs related to lung cancer and other lung diseases, and DAVID enrichment and functional analysis of every list. Table S2. Bioinformatic pipeline implemented in R. Table S3. Validation of expression levels of the most frequent differentially expressed lung cancer genes with TCGA RNA-Seq studies on the platform UALCAN. Table S4. Cytoscape common connection pattern analysis of all datasets, and Reactome FIViz analysis of signaling pathways and biological processes. Table S5. Gene regulatory networks analysis of NSCLC, SCLC, and PAH with the DAVID enrichment and functional analysis. Table S6. Transcription factor enrichment analysis by ChEA3 of the most frequent lung cancer DEGs. Table S7. Functional blocks of NSCLC, SCLC, and PAH gene regulatory networks.
