# Supplementary material for: Lung Cancer Gene Regulatory Network of Transcription Factors Related to the Hallmarks of Cancer
Source: Curr Issues Mol Biol. 2023 Jan 5;45(1):434–64. doi: 10.3390/cimb45010029 (PMC9857713; doi:10.3390/cimb45010029)
Supplement: Supplementary file 1 [file cimb-45-00029-s001.zip › Table S2.pdf]

```
# Lung cancer winning DEGs Expression matrix
setwd("~/")
eset1<-read.table("GSE19804_filtred.txt",h=T)
eset2<-read.table("GSE113439_normalized.txt",h=T)
eset3<-read.table("GSE108055_filtred.txt",h=T)
eset4<-read.table("GSE10072_filtred.txt",h=T)
eset5<-read.table("GSE52248_filtred.txt",h=T)
eset6<-read.table("GSE70089_filtred.txt",h=T)
eset7<-read.table("GSE81089_filtred.txt",h=T)
eset8<-read.table("GSE84776_filtred.txt",h=T)
eset9<-read.table("EMTAB3950_filtred.txt",h=T)
eset10<-read.table("EMTAB5231_filtred.txt",h=T)
eset11<-read.table("GSE3268_filtred.txt",h=T)
dim(eset…)
names(eset…)
head(eset…)

lista<-read.table("LISTLCWDEGs.txt")
lista<-read.table("LISTLCPAHWDEGs.txt")
lista<-read.table("LISTLCWTFS.txt")
lista<-as.character(lista$V1)
lista<-unique(lista)
length(lista)
class(lista)

todos1<-eset1$GENES
ind1<-which(todos1%in%lista)
length(ind1)
todos2<-eset2$GENES
ind2<-which(todos2%in%lista)
length(ind2)
todos3<-eset3$GENES
```

```
ind3<-which(todos3%in%lista)
length(ind3)
todos4<-eset4$GENES
ind4<-which(todos4%in%lista)
length(ind4)
todos5<-eset5$GENES
ind5<-which(todos5%in%lista)
length(ind5)
todos6<-eset6$GENES
ind6<-which(todos6%in%lista)
length(ind6)
todos7<-eset7$GENES
ind7<-which(todos7%in%lista)
length(ind7)
todos8<-eset8$GENES
ind8<-which(todos8%in%lista)
length(ind8)
todos9<-eset9$GENES
ind9<-which(todos9%in%lista)
length(ind9)
todos10<-eset10$GENES
ind10<-which(todos10%in%lista)
length(ind10)
todos11<-eset11$GENES
ind11<-which(todos11%in%lista)
length(ind11)

E1=eset1[ind1,]
E2=eset2[ind2,]
E3=eset3[ind3,]
E4=eset4[ind4,]
E5=eset5[ind5,]
```

```
E6=eset6[ind6,]
E7=eset7[ind7,]
E8=eset8[ind8,]
E9=eset9[ind9,]
E10=eset10[ind10,]
E11=eset11[ind11,]

dim(E…)
names(E…)
head(E…)

genes1<-E1$GENE
genes2<-E2$GENE
genes3<-E3$GENE
genes4<-E4$GENE
genes5<-E5$GENE
genes6<-E6$GENE
genes7<-E7$GENE
genes8<-E8$GENE
genes9<-E9$GENE
genes10<-E10$GENE
genes11<-E11$GENE

Em1<-as.matrix(E1[,-1])
dim(Em1)
rownames(Em1)<-genes1
Em2<-as.matrix(E2[,-1])
dim(Em2)
rownames(Em2)<-genes2
Em3<-as.matrix(E3[,-1])
dim(Em3)
rownames(Em3)<-genes3
```

```
Em4<-as.matrix(E4[,-1])
dim(Em4)
rownames(Em4)<-genes4
Em5<-as.matrix(E5[,-1])
dim(Em5)
rownames(Em5)<-genes5
Em6<-as.matrix(E6[,-1])
dim(Em6)
rownames(Em6)<-genes6
Em7<-as.matrix(E7[,-1])
dim(Em7)
rownames(Em7)<-genes7
Em8<-as.matrix(E8[,-1])
dim(Em8)
rownames(Em8)<-genes8
Em9<-as.matrix(E9[,-1])
dim(Em9)
rownames(Em9)<-genes9
Em10<-as.matrix(E10[,-1])
dim(Em10)
rownames(Em10)<-genes10
Em11<-as.matrix(E11[,-1])
dim(Em11)
rownames(Em11)<-genes11

#Gene expression correlation analysis of winning TFs
library(corrplot)
E1<-cor(t(Em1))
col3 <- colorRampPalette(c("blue", "white", "red"))
corrplot(E1,tl.cex=0.4,method="color",order="hclust", addrect=2, col=col3(20))
corrplot.mixed(E1,tl.cex=0.4)
heatmap(Em1,cexRow=0.4,cexCol = 0.4)
```

```
E3<-cor(t(Em3))

corrplot(E3,tl.cex=0.4,method="color",order="hclust", addrect=2, col=col3(20))

heatmap(Em3,cexRow=0.4,cexCol = 0.4)


# Construction of Coexpression networks and Common connectivity patterns

library(coexnet)

Net1 <- createNet(expData = Em1,threshold = 0.7,method = "correlation")

plot(Net1)

Net2 <- createNet(expData = Em2,threshold = 0.7,method = "correlation")

plot(Net2)

Net3 <- createNet(expData = Em3,threshold = 0.7,method = "correlation")

plot(Net3)

Net4 <- createNet(expData = Em4,threshold = 0.7,method = "correlation")

plot(Net4)

Net5 <- createNet(expData = Em5,threshold = 0.7,method = "correlation")

plot(Net5)

Net6 <- createNet(expData = Em6,threshold = 0.7,method = "correlation")

plot(Net6)

Net7 <- createNet(expData = Em7,threshold = 0.7,method = "correlation")

plot(Net7)

Net8 <- createNet(expData = Em8,threshold = 0.7,method = "correlation")

plot(Net8)

Net9 <- createNet(expData = Em9,threshold = 0.7,method = "correlation")

plot(Net9)

Net10 <- createNet(expData = Em10,threshold = 0.7,method = "correlation")

plot(Net10)

Net11 <- createNet(expData = Em11,threshold = 0.7,method = "correlation")

plot(Net11)


library(igraph)

write.graph(Net1,"~/Net19804LC8edges0.7.txt",format="ncol")
```

```
write.graph(Net2,"~/Net113439LC8edges0.7.txt",format="ncol")

write.graph(Net3,"~/Net108055LC8edges0.7.txt",format="ncol")

write.graph(Net4,"~/Net10072LC8edges0.7.txt",format="ncol")

write.graph(Net5,"~/Net52248LC8edges0.7.txt",format="ncol")

write.graph(Net6,"~/Net70089LC8edges0.7.txt",format="ncol")

write.graph(Net7,"~/Net81089LC8edges0.7.txt",format="ncol")

write.graph(Net8,"~/Net84776LC8edges0.7.txt",format="ncol")

write.graph(Net9,"~/Net3950LC8edges0.7.txt",format="ncol")

write.graph(Net10,"~/Net5231LC8edges0.7.txt",format="ncol")

write.graph(Net11,"~/Net3268LC8edges0.7.txt",format="ncol")


ccp <- CCP(Net1,Net2,Net3,Net4,Net5,Net6,Net7,Net8,Net9,Net10,Net11)

write.graph(ccp,"~/CCPLCPAHNETS.txt",format="ncol")

ccp2 <- CCP(Net1,Net3,Net4,Net5,Net6,Net7,Net8,Net9,Net10,Net11)

write.graph(ccp2,"~/CCPLCNETS.txt",format="ncol")

ccp3 <- CCP(Net1,Net2,Net3,Net4,Net9,Net10,Net11)

write.graph(ccp3,"~/CCPLCPAHMANETS.txt",format="ncol")

ccp4 <- CCP(Net2,Net5,Net6,Net7,Net8)

write.graph(ccp4,"~/CCPLCPAHRNASEQNETS.txt",format="ncol")

ccp5 <- CCP(Net1,Net3,Net4,Net9,Net10,Net11)

write.graph(ccp5,"~/CCPLCMANETS.txt",format="ncol")

ccp6 <- CCP(Net5,Net6,Net7,Net8)

write.graph(ccp6,"~/CCPLCRNASEQNETS.txt",format="ncol")


#Gedevo aligments

gedevo.alignment<-
function(network1,network2,type.file="edgelist",use.names=T,max.iterations=100,limit.time=5,iterations.converge=25,random
ize=F){


  name1<-as.character(substitute(network1))

  name2<-as.character(substitute(network2))


  require(igraph)
```

```
require(orca)


### FUNCIONES ###

pesos.orbitas<-function(){

  O<-
c(1,2,2,2,3,4,3,3,3,3,4,4,4,4,3,4,6,5,4,5,6,6,4,4,5,5,8,4,6,6,7,5,6,6,6,5,6,7,7,5,7,7,7,6,5,5,6,8,8,6,6,8,6,9,6,6,4,6,6,8,9,6,6,8,8,6,7,7,8,5,6,6,4
) #Número de graphlets de cada orbita (necesaria para los pesos)

  pesos<-1-(log(O)/log(73))


  return(pesos)

}

lectura.redes<-function(data1,data2,tipo){


  if(tipo=="edgelist"){

    red1<-graph.data.frame(data1,directed=F)

    red2<-graph.data.frame(data2,directed=F)

  }


  if(tipo=="adjacency"){

    red1<-graph.adjacency(as.matrix(data1),mode="undirected",add.colnames=NULL,diag=FALSE)

    red2<-graph.adjacency(as.matrix(data2),mode="undirected",add.colnames=NULL,diag=FALSE)

  }


  if(tipo=="igraph"){

    red1<-as.undirected(data1)

    red2<-as.undirected(data2)

  }


  cambio=F

  if(length(V(red1))<length(V(red2))){ red<-red1 ;red1<-red2 ; red2<-red;remove(red) ; cambio=T }


  redes<-list("Red1"=red1,"Red2"=red2,"Cambio"=cambio)
```

```
  return(redes)
}
poblacion.inicial<-function(red1,red2,nombres,init.rand,pesos.grlets){

  #Vector con los nodos de cada una de las redes
  nodes1<-names(V(red1))
  nodes2<-names(V(red2))

  if(init.rand==TRUE){

    # En caso de que la segunda red sea más pequeña se agregan "nodos vacios"
    if(length(nodes1)>length(nodes2)){nodes2<-c(nodes2,rep(NA,length(nodes1)-length(nodes2)))}

    if(nombres==TRUE){

      iguales<-nodes1[!is.na(factor(nodes1,levels=nodes2))]
      iguales<-data.frame("net1"=iguales,"net2"=iguales)

      dif1<-nodes1[is.na(factor(nodes1,levels=nodes2))]
      dif2<-nodes2[is.na(factor(nodes2,levels=nodes1))]
      alineamiento<-data.frame("net1"=dif1[sample.int(length(dif1))],
                               "net2"=dif2[sample.int(length(dif2))])

      alineamiento<-rbind(iguales,alineamiento)

    }else{
      alineamiento<-data.frame("net1"=nodes1[sample.int(length(nodes1))],
                               "net2"=nodes2[sample.int(length(nodes2))])
    }

    alineamiento<-subset(alineamiento,!is.na(alineamiento$net2)) #Se eliminan los nodos que no estan alineados con otro
  }
```

```
if(init.rand==FALSE){
 if(nombres==TRUE){

  iguales<-nodes1[!is.na(factor(nodes1,levels=nodes2))]
  iguales<-data.frame("net1"=iguales,"net2"=iguales)

  if(nrow(iguales)<length(nodes2)){

   dif1<-nodes1[is.na(factor(nodes1,levels=nodes2))]
   dif2<-nodes2[is.na(factor(nodes2,levels=nodes1))]
   all_align<-data.frame("net1"=rep(dif1,each=length(dif2)),
                         "net2"=rep(dif2,times=length(dif1)))

   alineamiento<-data.frame()
   for (i in 1:length(dif2)) {
    a<-subset(all_align,all_align$net2==dif2[i])
    a$grlets<-grlets(a,red1,red2,pesos.grlets)
    alineamiento<-rbind(alineamiento,a[which.max(a$grlets),])
    all_align<-subset(all_align,all_align$net1!=alineamiento[i,1] & all_align$net2!=alineamiento[i,2])
   }

   alineamiento<-rbind(iguales,alineamiento[,c("net1","net2")])
  }else{
   alineamiento<-iguales
  }

 }else{

  all_align<-data.frame("net1"=rep(nodes1,each=length(nodes2)),
                        "net2"=rep(nodes2,times=length(nodes1)))
```

```
    alineamiento<-data.frame()
    for (i in 1:length(nodes2)) {
      a<-subset(all_align,all_align$net2==nodes2[i])
      a$grlets<-grlets(a,red1,red2,pesos.grlets)
      alineamiento<-rbind(alineamiento,a[which.max(a$grlets),])
      all_align<-subset(all_align,all_align$net1!=alineamiento[i,1] & all_align$net2!=alineamiento[i,2])
    }

    alineamiento<-alineamiento[,c("net1","net2")]
  }
 }

 alineamiento$net1<-as.character(alineamiento$net1);alineamiento$net2<-as.character(alineamiento$net2)
 alineamiento$align<-paste(alineamiento$net1,alineamiento$net2,sep=" -- ")
 alineamiento$iter<-0 #Inicializar Nro de iteraciones
 alineamiento$health<-100 #Inicializar salúd

 return(alineamiento)
}
GED<-function(alineamiento,red1,red2){

 ged_alignment<-rep(0,nrow(alineamiento))
 for (i in 1:nrow(alineamiento)) {

   #Nodos vecinos de los nodos que se están comparando
   neigh1<-names(neighbors(red1,alineamiento$net1[i]))
   neigh2<-names(neighbors(red2,alineamiento$net2[i]))

   #Se identifica el nodo que tiene un vecindario más pequeño
   if(length(neigh1)<=length(neigh2)){
     small=neigh1;big=neigh2
     nsmall<-"net1";nbig<-"net2"
```

```
    }else{
      small=neigh2;big=neigh1
      nsmall<-"net2";nbig<-"net1"
    };remove(neigh1,neigh2)

    #Se crea un contador que ira aumentando cada vez que encuentre vecinos alineados
    ged=0
    for (j in 1:length(small)){
      vecino.igual=length(which(big==alineamiento[,nbig][alineamiento[,nsmall]==small[j]]))
      if(vecino.igual==1){ged=ged+1}
    }

    #Finalmente, con el contador de vecinos alineados se calcula GED(u,v) de la siguiente manera:
    #(Tamaño del vecindario grande - vecinos alineados) + (nodos a crear en vecindario pequeño para tener el mismo tamaño del vecindario grande)
    ged=(length(big)-ged)+(length(big)-length(small))
    ged=ged/((length(big)*2)-1) #Se hace esto para que esté en un intervalo de [0,1]

    ged_alignment[i]<-ged
  }

  return(ged_alignment)
}
grlets<-function(alineamiento,red1,red2,pesos){

  #Tenemos que crear una gran red con el alineamiento (sera necesario renombrar por si los nodos tienen el mismo nombre)
  edgelist1<-as_edgelist(red1)
  edgelist1[,1]<-paste(edgelist1[,1],"-net1",sep="");edgelist1[,2]<-paste(edgelist1[,2],"-net1",sep="")

  edgelist2<-as_edgelist(red2)
  edgelist2[,1]<-paste(edgelist2[,1],"-net2",sep="");edgelist2[,2]<-paste(edgelist2[,2],"-net2",sep="")
```

```
    # Aquí surgio la duda de que si además de deben unis en la red los nodos que se están alineando, en ese caso se haria con similares:

    similares<-matrix(c(alineamiento$net1,alineamiento$net2),nrow(alineamiento),2)

    similares[,1]<-paste(similares[,1],"-net1",sep="");similares[,2]<-paste(similares[,2],"-net2",sep="")

    #Se unen las tres redes: red1, red2 y alineamiento

    align_net<-rbind(edgelist1,edgelist2);remove(edgelist1,edgelist2,similares)


    #Para la funcion count5 será necesario un grafo con nombres de nodos 1,2,..., se guarda la correspondencia con los nombres originales

    corres<-data.frame("V"=names(V(graph.data.frame(align_net,directed = F))),

                   "ID"=seq(1,length(names(V(graph.data.frame(align_net,directed = F)))),1))


    #Se reemplazan los nombres de nodos por números enteros

    for (i in 1:nrow(corres)) {

      align_net[align_net==corres$V[i]]<-corres$ID[i]

    }

    align_net<-matrix(as.integer(align_net),nrow(align_net),2)


    #Cálculo de las orbitas de la gran red

    orb<-count5(align_net)


    #Ahora se calculará signature para cada par de nodos alineados

    s_alignment<-rep(0,nrow(alineamiento))

    for (i in 1:nrow(alineamiento)) {

      u=corres$ID[corres$V==paste(alineamiento$net1[i],"-net1",sep="")]

      v=corres$ID[corres$V==paste(alineamiento$net2[i],"-net2",sep="")]


      D=pesos*(abs(log(orb[u,]+1)-log(orb[v,]+1))/log(max(orb[u,],orb[v,])+2))

      s_alignment[i]=1-(sum(D)/sum(pesos))

    }


    return(s_alignment)

  }
```

```
salud<-function(alineamiento){

  bi<-median(alineamiento$Score)+(IQR(alineamiento$Score)/2)
  bi<-length(which(alineamiento$Score>=bi))

  salud<-alineamiento$health

  if(bi>0 & bi<nrow(alineamiento)){
    Pos.Score<-seq(bi+1,nrow(alineamiento),1)/nrow(alineamiento) # Posición/Tamaño de población
    GED<-alineamiento$GED[-c(1:bi)]
    iter<-alineamiento$iter[-c(1:bi)]

    salud[-c(1:bi)]=salud[-c(1:bi)]-((Pos.Score+GED)/(2*((iter+1)^2)))
  }else if(bi==nrow(alineamiento)){
    salud=salud
  }else if(bi==0){
    Pos.Score<-seq(bi+1,nrow(alineamiento),1)/nrow(alineamiento) # Posición/Tamaño de población
    salud=salud-((Pos.Score+alineamiento$GED)/(2*((alineamiento$iter+1)^2)))
  }

  return(salud)
}
muerte<-function(alineamiento){

  nd<-median(alineamiento$health)-(IQR(alineamiento$health)/2)
  if(nd<min(alineamiento$healt)){
    nd=quantile(alineamiento$health,0.05)
  }
  nd<-length(which(alineamiento$health<nd))

  if(nd>0){
    alineamiento<-alineamiento[order(alineamiento$health,decreasing=T),] #Ordenar en base a la salúd
```

```
    alineamiento<-alineamiento[-c((nrow(alineamiento)-nd+1):nrow(alineamiento)),] #Eliminar los mas "enfermos"
  }

  ME<-list("Alineamiento"=alineamiento,"NM"=nd)

  return(ME)
}
mutar<-function(alineamiento,red1,red2,muertos,peores,pesos.grlets){
  #MUTACIÓN (generar nuevos individuos)

  alineamiento<-alineamiento[order(alineamiento$Score,decreasing=T),]
  mut<-data.frame() #Tabla donde se guardan las posibles mutaciones

  if(peores>0){

    for (i in 1:round(nrow(alineamiento)/1)) {

      #Vecinos por cada padre
      neigh1<-names(neighbors(red1,alineamiento$net1[i]))
      neigh2<-names(neighbors(red2,alineamiento$net2[i]))

      #Igualar la dimensión de los vectores de vecinos
      if(length(neigh1)>length(neigh2)){
        neigh2<-c(neigh2,rep(NA,length(neigh1)-length(neigh2)))
      }else{
        neigh1<-c(neigh1,rep(NA,length(neigh2)-length(neigh1)))
      }

      #Aleatorizar cruze entre vecinos de los padres
      mut1<-data.frame("net1"=neigh1[sample.int(length(neigh1))],
                       "net2"=neigh2[sample.int(length(neigh2))])
      mut<-rbind(mut,mut1) #Ir pegando las mutaciones de cada pareja
```

```
}
mut<-subset(mut,!is.na(mut$net2));mut<-subset(mut,!is.na(mut$net1)) #Eliminar nodos alineados con NA


#nodos que aún no están alineados actualmente
nodes1<-names(V(red1))[(names(V(red1)) %in% alineamiento$net1)==F]
nodes2<-names(V(red2))[(names(V(red2)) %in% alineamiento$net2)==F]
# En caso de que la segunda red sea más pequeña se agregan "nodos vacios"
if(length(nodes1)>length(nodes2)){nodes2<-c(nodes2,rep(NA,length(nodes1)-length(nodes2)))}


mut1<-data.frame("net1"=nodes1[sample.int(length(nodes1))],
                 "net2"=nodes2[sample.int(length(nodes2))])
mut1<-subset(mut1,!is.na(mut1$net2)) #Se eliminan los nodos que no estan alineados con otro
mut<-rbind(mut,mut1);remove(mut1)


mut$align<-paste(mut$net1,mut$net2,sep=" -- ")
mut<-mut[!duplicated(mut$align),]


mut<-subset(mut,(mut$net1 %in% alineamiento$net1)==F)
mut<-subset(mut,(mut$net2 %in% alineamiento$net2)==F)


mut<-mut[sample.int(nrow(mut)),]
mut<-mut[!duplicated(mut$net1),];mut<-mut[!duplicated(mut$net2),]


c=0;maximo=length(V(red2))*10
while(nrow(mut)<peores){
  mut1<-data.frame("net1"=nodes1[sample.int(length(nodes1))],
                   "net2"=nodes2[sample.int(length(nodes2))])
  mut1$align<-paste(mut1$net1,mut1$net2,sep=" -- ")
  mut1<-subset(mut1,!is.na(mut1$net2)) #Se eliminan los nodos que no estan alineados con otro
  mut<-rbind(mut,mut1);remove(mut1)


  mut$align<-paste(mut$net1,mut$net2,sep=" -- ")
```

```
      mut<-mut[!duplicated(mut$align),]


      mut<-subset(mut,(mut$net1 %in% alineamiento$net1)==F)
      mut<-subset(mut,(mut$net2 %in% alineamiento$net2)==F)


      mut<-mut[sample.int(nrow(mut)),]
      mut<-mut[!duplicated(mut$net1),];mut<-mut[!duplicated(mut$net2),]


      c=c+1
      if(c==maximo){break}
    }


    score<-grlets(mut,red1,red2,pesos.grlets)
    mut<-mut[order(score,decreasing=T),]
    mut<-mut[1:peores,]
    mut$iter<-0
    mut$health<-100
    mut$GED<-NA
    mut$grlets<-NA
    mut$Score<-NA
  }


  return(mut)
}
gedevo<-function(net1,net2,nmbrs,rndm,max.iter,max.time,convergence){


  #Inicicalizar parametros importantes
  W<-pesos.orbitas()
  iter=0;algorithm.time=0;cnvrg=0
  start.time=Sys.time()


  #Generar población inicial (opción de emparejar por nombres)
```

```
  alignment<-poblacion.inicial(net1,net2,nombres=nmbrs,init.rand=rndm,pesos.grlets=W)
  past.better<-alignment$align

  while(iter<max.iter & algorithm.time<=max.time & cnvrg<convergence){

    ##### GED #####
    alignment$GED<-GED(alignment,net1,net2) #Un valor entre 0 y 1, donde 0 indica que son similares
    ##### Signatures #####
    alignment$grlets<-grlets(alignment,net1,net2,W) #Un valor entre 0 y 1, donde 1 indica que son similares
    ##### PairScore #####
    alignment$Score<-((1-alignment$GED)*(2/3))+(alignment$grlets*(1/3)) #Un valor entre 0 y 1, donde 1 indica que son similares

    ##### Salud #####
    alignment<-alignment[order(alignment$Score,decreasing=T),] #Ordenar en base al PairScore
    alignment$health<-salud(alignment)
    ##### Muerte #####
    death<-muerte(alignment)
    alignment<-death$Alineamiento
    ##### Convergencia #####
    if(length(which((alignment$align %in% past.better)==TRUE))==min(nrow(alignment),length(past.better))){
      cnvrg=cnvrg+1
    }else{
      cnvrg=0
    }
    past.better<-alignment$align
    ##### Mutación y Descendencia #####
    new<-mutar(alignment,net1,net2,dead,death$NM,W)
    ##### Nueva población #####
    alignment$iter=alignment$iter+1
    alignment<-rbind(alignment,new)
```

```
    iter=iter+1

    algorithm.time=difftime(Sys.time(),start.time,units="min")


    if(length(new$net1[is.na(new$net1)])>0){alignment<-subset(alignment,!is.na(alignment$align))}

  }


  if(length(alignment$Score[is.na(alignment$Score)])>0){

    alignment$GED<-GED(alignment,net1,net2) #Un valor entre 0 y 1, donde 0 indica que son similares

    ##### Signatures #####

    alignment$grlets<-grlets(alignment,net1,net2,W) #Un valor entre 0 y 1, donde 1 indica que son similares

    ##### PairScore #####

    alignment$Score<-((1-alignment$GED)*(2/3))+(alignment$grlets*(1/3)) #Un valor entre 0 y 1, donde 1 indica que son
similares

  }


  alignment<-alignment[order(alignment$Score,decreasing=T),]


  objets=list("Alignment"=alignment,

              "Time"=paste(round(as.numeric(algorithm.time),4),"minutes",sep=" "),

              "Convergence"=cnvrg,

              "Iterations"=iter)


  return(objets)

}


#Leer las redes y convertirlas en un objeto de igraph

networks<-lectura.redes(network1,network2,tipo=type.file)

#Correr el algoritmo de gedevo

result<-gedevo(networks$Red1,networks$Red2,use.names,randomize,max.iterations,limit.time,iterations.converge)


if(networks$Cambio==F){

  names(result$Alignment)[1:2]<-c(name1,name2)
```

```
  }else{
    names(result$Alignment)[1:2]<-c(name2,name1)
  }

  result<-list("alignment"=result$Alignment[,c(1:3,6:8)],
                              "network1"=networks$Red1,
               "network2"=networks$Red2,
               "time"=result$Time,
               "convergence"=result$Convergence,
               "iterations"=result$Iterations,
               "detailed_alignment"=result$Alignment)

  return(result)
}

#Contruction of gene regulatory and coregulatory networks
library("CoRegNet")
grn = hLICORN(Em1, TFlist=TFlist)
grn = hLICORN(Em3, TFlist=TFlist)
influence = regulatorInfluence(grn,Em1)
influence = regulatorInfluence(grn,Em3)
coregs = coregulators(grn)
write.table(coregs,"GSE108055SCLPAHCOREGS.txt")
write.table(coregs,"GSE108055SCLCCOREGS.txt")
write.table(coregs,"GSE19804NSCLCPAHCOREGS.txt")
write.table(coregs,"GSE19804NSCLCCOREGS.txt")
display(grn,Em1,influence)
display(grn,Em3,influence)
print(grn)
Rnet <- coregnetToDataframe(grn)
write.table(Rnet,"GSE108055SCLPAHGRN.txt")
write.table(Rnet,"GSE108055SCLCGRN.txt")
```

```
write.table(Rnet,"GSE19804NSCLCPAHGRN.txt")

write.table(Rnet,"GSE19804NSCLCGRN.txt")


#Construction of fibers or functional blocks

library(fibrationSymmetries)

setwd("~/FibrationSymmetries")

$Get building block classification and put the building block summary in the pdf format in the "buildingBlocks" folder

buildingBlocks = get.building.blocks(file = "NSCLCPAHTFS.txt", sep = ",", outputFolder = "NSCLCESTBuildingBlocks", pdf = T)

buildingBlocks = get.building.blocks(file = "NSCLCFS.txt", sep = ",", outputFolder = "NSCLCPROGBuildingBlocks", pdf = T)

buildingBlocks = get.building.blocks(file = "SCLCPAHTFS.txt", sep = ",", outputFolder = "SCLCESTBuildingBlocks", pdf = T)

buildingBlocks = get.building.blocks(file = "SCLCFS.txt", sep = ",", outputFolder = "SCLCPROGBuildingBlocks", pdf = T)


#Construction of transcriptional regulatory networks of winning transcription factors

library(RTN)

TFlist <-c("SOX4","BZW2","FOXM1","ZBTB16","TAL1","SOX17","KLF4")

RTN <- tni.constructor(expData = Em1, regulatoryElements = TFlist)

RTN <- tni.constructor(expData = Em3, regulatoryElements = TFlist)

rtni <- tni.permutation(RTN, nPermutations = 100)

rtni <- tni.bootstrap(rtni)

rtni <- tni.dpi.filter(rtni)

tni.regulon.summary(rtni)

tni.regulon.summary(rtni, regulatoryElements = "SOX17")

tni.regulon.summary(rtni, regulatoryElements = "SOX4")

regulons <- tni.get(rtni, what = "regulons.and.mode", idkey = "ID")

head(regulons$SOX17)

head(regulons$SOX4)

g <- tni.graph(rtni, regulatoryElements = c("SOX4","BZW2","FOXM1","ZBTB16","TAL1","SOX17","KLF4"))

library(RedeR)

rdp <- RedPort()

calld(rdp)

addGraph(rdp, g, layout=NULL)
```

```
addLegend.color(rdp, g, type="edge")

addLegend.shape(rdp, g)

relax(rdp, ps = TRUE)
```
