# Supplementary material for: Lung Cancer Gene Regulatory Network of Transcription Factors Related to the Hallmarks of Cancer
Source: Curr Issues Mol Biol. 2023 Jan 5;45(1):434–64. doi: 10.3390/cimb45010029 (PMC9857713; doi:10.3390/cimb45010029)
Supplement: Supplementary file 1 [file cimb-45-00029-s001.zip › Table S3.pdf]

DOWN	UALCAN-TCGA-LUAD	UALCAN-TCGA-LUSC	UP	UALCAN-TCGA-LUAD	UALCAN-TCGA-LUSC
ABCA8	1.62447832963153E-12	1.62447832963153E-12	AARS	1.62436730732907E-12	<1E-12
ACACB	1.62470037423645E-12	<1E-12	ABCA12	1.62447832963153E-12	<1E-12
ACADL	1.62447832963153E-12	1.62447832963153E-12	ACTL6A	<1E-12	1.62447832963153E-12
ACTG2	1.41389999708252E-09	5.28610000216645E-08	ALDH18A1	1.62447832963153E-12	1.62436730732907E-12
ACTN2	3.33066907387547E-16	1.62447832963153E-12	APEX1	<1E-12	1.62458935193399E-12
ACVRL1	<1E-12	1.62447832963153E-12	ASF1B	1.62436730732907E-12	<1E-12
ADAMTS8	<1E-12	1.62447832963153E-12	ASPM	<1E-12	1.62447832963153E-12
ADAMTSL3	<1E-12	1.62569957495862E-12	AURKA	1.62447832963153E-12	<1E-12
ADH1B	1.62436730732907E-12	1.62447832963153E-12	AURKB	<1E-12	<1E-12
ADRB1	2.57571741713036E-13	3.26759952429256E-10	B4GALT2	<1E-12	<1E-12
ADRB2	1.62447832963153E-12	1.62436730732907E-12	BIRC5	1.62436730732907E-12	<1E-12
AGTR1	1.62447832963153E-12	<1E-12	BUB1	<1E-12	1.62447832963153E-12
ANGPT1	1.62447832963153E-12	1.62625468647093E-12	BUB1B	1.62447832963153E-12	1.62447832963153E-12
AOC3	<1E-12	1.62447832963153E-12	BZW2	1.62447832963153E-12	1.62436730732907E-12
AOX1	8.45350000000522E-05	5.81799999954669E-07	CA9	1.6242562850266E-12	<1E-12
AQP4	1.62447832963153E-12	<1E-12	CAD	1.62436730732907E-12	1.62447832963153E-12
ARHGAP6	1.62447832963153E-12	1.62447832963153E-12	CBLC	1.62436730732907E-12	<1E-12
ASPA	<1E-12	1.62447832963153E-12	CCNA2	<1E-12	1.62447832963153E-12
BMP5	8.48270000020079E-07	1.62447832963153E-12	CCNB1	<1E-12	1.62436730732907E-12
C1QTNF7	<1E-12	1.62447832963153E-12	CCNB2	1.62447832963153E-12	<1E-12
CA2	6.84479994905729E-09	3.58480023443519E-10	CCNE1	<1E-12	1.62436730732907E-12
CA4	1.62436730732907E-12	1.62436730732907E-12	CCNF	<1E-12	<1E-12
CAB39L	<1E-12	1.62447832963153E-12	CCT3	1.62447832963153E-12	<1E-12
CASQ2	8.14880385391348E-11	9.29709997876671E-09	CDC20	1.62447832963153E-12	1.62447832963153E-12
CAV1	<1E-12	<1E-12	CDC6	<1E-12	1.62447832963153E-12
CD33	<1E-12	<1E-12	CDCA3	1.62458935193399E-12	1.62447832963153E-12
CD36	<1E-12	1.62447832963153E-12	CDCA4	<1E-12	1.62447832963153E-12
CDH5	<1E-12	1.62447832963153E-12	CDCA8	1.62436730732907E-12	1.11022302462516E-16
CDO1	<1E-12	1.62458935193399E-12	CDKN3	<1E-12	<1E-12
CFD	<1E-12	1.62447832963153E-12	CENPA	<1E-12	1.62458935193399E-12
CHRDL1	1.62458935193399E-12	<1E-12	CENPE	1.62436730732907E-12	1.62447832963153E-12
CLDN18	1.62447832963153E-12	1.62447832963153E-12	CENPF	1.62447832963153E-12	<1E-12
CLEC1A	<1E-12	<1E-12	CENPM	<1E-12	<1E-12
CLIC5	<1E-12	1.62436730732907E-12	CENPO	1.62447832963153E-12	1.62447832963153E-12
CNN1	2.67179611768142E-11	1.24340004958867E-09	CEP55	1.62436730732907E-12	<1E-12
CORO2B	3.66373598126302E-15	1.62447832963153E-12	CHAF1A	1.62436730732907E-12	<1E-12
CPA3	7.65054686269195E-13	1.6251444634463E-12	CHEK1	<1E-12	<1E-12
CXCL12	1.6242562850266E-12	1.6251444634463E-12	CHEK2	1.62447832963153E-12	<1E-12
CYB5A	<1E-12	1.62447832963153E-12	CIT	1.62447832963153E-12	3.52380347123926E-11
DENND2A	1.62436730732907E-12	<1E-12	CKAP4	<1E-12	<1E-12
DES	1.63435931455069E-12	7.75968178601261E-12	COL10A1	<1E-12	1.62447832963153E-12
DNAI1	1.403190E-03	1.75588999999921E-05	COL11A1	1.6242562850266E-12	<1E-12
DUOX1	1.62447832963153E-12	2.44249065417534E-15	COL5A2	<1E-12	<1E-12
DUSP1	1.53210777398272E-13	<1E-12	CYC1	<1E-12	<1E-12

EFCAB1	1.445050E-04	6.49320000000175E-05	DBN1	1.62458935193399E-12	1.62447832963153E-12
EMCN	<1E-12	1.62436730732907E-12	DEPDC1	1.62447832963153E-12	1.62458935193399E-12
ENG	1.62481139653892E-12	<1E-12	DLGAP5	<1E-12	<1E-12
EPAS1	1.62447832963153E-12	1.62503344114384E-12	DLX5	1.69348999999874E-05	2.44249065417534E-15
F8	4.62196947381699E-12	1.62447832963153E-12	DSG2	<1E-12	1.62436730732907E-12
FABP4	1.62503344114384E-12	1.62458935193399E-12	DTL	1.62436730732907E-12	1.62447832963153E-12
FAM107A	2.37665442881507E-12	5.0003001739185E-11	EIF2AK1	1.62436730732907E-12	<1E-12
FAM184A	1.62447832963153E-12	1.62447832963153E-12	EIF4EBP1	1.62447832963153E-12	<1E-12
FBLN5	1.62436730732907E-12	1.62447832963153E-12	ENO2	<1E-12	<1E-12
FCN3	2.06878958408652E-12	1.0170975173196E-11	ERCC6L	<1E-12	<1E-12
FGL2	4.25029999999715E-05	1.62447832963153E-12	ESPL1	<1E-12	<1E-12
FGR	1.62458935193399E-12	<1E-12	EXO1	1.62436730732907E-12	1.62447832963153E-12
FHL1	<1E-12	1.62447832963153E-12	FANCI	<1E-12	<1E-12
FHL5	<1E-12	1.62447832963153E-12	FEN1	<1E-12	1.62447832963153E-12
FMO2	1.62447832963153E-12	4.44089209850063E-16	FERMT1	<1E-12	1.62436730732907E-12
FOLR1	3.62154750632726E-13	1.62447832963153E-12	FKBP4	<1E-12	<1E-12
FOXF1	1.62481139653892E-12	1.62447832963153E-12	FOXM1	1.62447832963153E-12	<1E-12
FZD4	3.92630372658687E-12	1.62470037423645E-12	GINS1	<1E-12	1.62447832963153E-12
GIMAP6	1.62436730732907E-12	1.62447832963153E-12	GINS2	<1E-12	1.62447832963153E-12
GIPC2	1.6242562850266E-12	1.62436730732907E-12	GINS3	<1E-12	<1E-12
GNG11	1.81743509131138E-12	<1E-12	GMNN	1.62447832963153E-12	1.62447832963153E-12
GPD1	1.62447832963153E-12	1.62447832963153E-12	GORASP2	<1E-12	1.62436730732907E-12
GPD1L	<1E-12	1.62447832963153E-12	GPI	<1E-12	<1E-12
GPM6A	1.62447832963153E-12	1.62447832963153E-12	GPX2	<1E-12	<1E-12
GPX3	1.62458935193399E-12	1.62458935193399E-12	GREM1	1.62447832963153E-12	1.62436730732907E-12
GYPC	1.62436730732907E-12	1.62447832963153E-12	HJURP	1.62447832963153E-12	<1E-12
HBB	9.12340003544898E-09	7.00069999748365E-08	HMMR	1.62436730732907E-12	<1E-12
HIGD1B	<1E-12	1.62447832963153E-12	HOXC6	<1E-12	<1E-12
HLF	4.44089209850063E-16	<1E-12	HPRT1	<1E-12	<1E-12
IFT57	1.74704695155015E-12	<1E-12	KIF11	1.62436730732907E-12	1.62447832963153E-12
IGSF10	<1E-12	1.62525548574877E-12	KIF14	1.62447832963153E-12	<1E-12
IL18RAP	5.789500E-04	2.00889999999454E-05	KIF15	1.62447832963153E-12	<1E-12
IL33	1.62447832963153E-12	1.6251444634463E-12	KIF20A	<1E-12	1.62447832963153E-12
ITGA8	1.76281211849982E-12	1.6538992397841E-12	KIF23	<1E-12	<1E-12
ITIH5	1.62447832963153E-12	<1E-12	KIF2C	<1E-12	<1E-12
ITM2A	3.33066907387547E-16	<1E-12	KIF4A	<1E-12	<1E-12
JAM2	1.62458935193399E-12	1.62447832963153E-12	KIFC1	1.62436730732907E-12	<1E-12
KCNA5	2.09220000213506E-08	1.61339996918031E-09	LAPTM4B	<1E-12	1.62458935193399E-12
KLF2	2.29980479105052E-11	1.62592161956354E-12	LMNB1	1.62447832963153E-12	1.62447832963153E-12
KLF4	4.45532499782075E-13	2.57889998245275E-09	LMNB2	<1E-12	1.62447832963153E-12
LDB2	1.62447832963153E-12	1.62447832963153E-12	LRP8	1.62458935193399E-12	<1E-12
LEPR	1.63868918434673E-12	7.03470615093238E-12	LSR	<1E-12	<1E-12
LIMS2	<1E-12	1.62447832963153E-12	MAD2L1	1.62447832963153E-12	<1E-12
LMO2	<1E-12	1.62447832963153E-12	MARCKSL1	1.62447832963153E-12	<1E-12
LMOD1	2.12130313315129E-12	1.73061565078569E-12	MCM10	<1E-12	1.62447832963153E-12

LRRC32	3.99469346490378E-12	1.09249997937155E-09	MCM2	1.62447832963153E-12	1.6242562850266E-12
LRRC36	1.62514446344463E-12	1.62914126633495E-12	MCM3	<1E-12	1.62447832963153E-12
LTBP4	1.62514446344463E-12	1.13242748511766E-14	MCM4	<1E-12	<1E-12
LYVE1	1.67232894199287E-12	1.76991754585742E-12	MCM6	<1E-12	1.62447832963153E-12
MAMDC2	1.62458935193399E-12	1.62447832963153E-12	MCM7	<1E-12	<1E-12
MEIS1	3.34021699188725E-12	6.47659999999384E-05	MELK	<1E-12	<1E-12
MFAP4	1.62447832963153E-12	<1E-12	MKI67	1.62436730732907E-12	<1E-12
MGP	1.33639765920179E-11	1.62436730732907E-12	MMP1	1.83170001388788E-09	1.62436730732907E-12
MMRN1	9.97409932423921E-11	3.10651504520365E-12	MMP11	<1E-12	1.62447832963153E-12
MMRN2	<1E-12	1.62447832963153E-12	MMP14	<1E-12	1.62447832963153E-12
MNDA	1.62436730732907E-12	<1E-12	MTHFD2	<1E-12	<1E-12
MYCT1	1.62436730732907E-12	1.62447832963153E-12	MYBL2	<1E-12	<1E-12
MYH10	2.20279350315877E-12	3.64375196681976E-13	NCAPG	<1E-12	1.62458935193399E-12
MYL9	1.62436730732907E-12	1.62436730732907E-12	NDC80	1.62436730732907E-12	<1E-12
NR2F1	7.05889999785825E-08	1.62547753035369E-12	NEK2	<1E-12	1.62447832963153E-12
NR4A3	9.6573000041289E-08	1.68370000053208E-07	NETO2	1.62447832963153E-12	1.11022302462516E-16
PALMD	<1E-12	1.62458935193399E-12	NIPSNAP1	1.62447832963153E-12	1.62436730732907E-12
PCOLCE2	<1E-12	1.62447832963153E-12	NME4	1.62447832963153E-12	<1E-12
PDE2A	6.58729959468474E-10	2.98779889718048E-11	NUF2	1.62447832963153E-12	<1E-12
PDE5A	<1E-12	1.62581059726108E-12	NUSAP1	1.62447832963153E-12	1.62436730732907E-12
PDE8B	3.99680288865056E-14	1.62525548574877E-12	OIP5	1.62447832963153E-12	<1E-12
PDK4	8.84709971948894E-10	2.60720334210873E-11	PAICS	<1E-12	<1E-12
PDZD2	<1E-12	1.62447832963153E-12	PAK6	<1E-12	<1E-12
PIK3R1	2.54440912783593E-12	1.40150000005335E-06	PBK	1.62447832963153E-12	1.62447832963153E-12
PIP5K1B	1.62447832963153E-12	1.62447832963153E-12	PCNA	1.11022302462516E-16	<1E-12
PKNOX2	<1E-12	1.62447832963153E-12	PDIA6	<1E-12	<1E-12
PLAC8	2.96600521920709E-11	2.09179999988152E-07	PLK1	1.62447832963153E-12	<1E-12
PLCE1	1.63624669369256E-12	1.65623070813581E-12	PLOD2	1.62447832963153E-12	<1E-12
PLLP	1.62447832963153E-12	1.62447832963153E-12	PPAT	1.62458935193399E-12	<1E-12
PODXL	1.62436730732907E-12	1.62447832963153E-12	PRAME	4.44089209850063E-16	<1E-12
PRX	1.63025148935958E-12	2.35522712443981E-12	PRC1	<1E-12	1.62447832963153E-12
PTGER4	1.62436730732907E-12	<1E-12	PRPF19	1.62436730732907E-12	<1E-12
PZP	1.716050E-03	4.98640000001771E-06	PSAT1	1.62447832963153E-12	<1E-12
RAB11FIP1	1.62447832963153E-12	1.62436730732907E-12	PSMD2	<1E-12	1.62447832963153E-12
RAI2	1.62447832963153E-12	<1E-12	PTTG1	1.62458935193399E-12	<1E-12
RAMP3	1.64168678651322E-12	2.52076137741142E-12	RACGAP1	<1E-12	<1E-12
RASIP1	<1E-12	1.62458935193399E-12	RAD51AP1	1.62436730732907E-12	<1E-12
RBMS3	5.55111512312578E-16	1.62492241884138E-12	RAD54L	1.62447832963153E-12	1.62436730732907E-12
RGN	1.62625468647093E-12	1.62436730732907E-12	RANGAP1	1.62447832963153E-12	1.62447832963153E-12
ROBO4	1.62447832963153E-12	1.62447832963153E-12	RCC1	<1E-12	<1E-12
RSPO1	8.32667268468867E-15	1.6068035790795E-11	RFC2	<1E-12	1.62447832963153E-12
S1PR1	1.62447832963153E-12	1.62447832963153E-12	RFC4	<1E-12	<1E-12
SAMHD1	1.6242562850266E-12	1.62481139653892E-12	RFWD3	1.6242562850266E-12	<1E-12
SCGB1A1	9.73829999972864E-07	2.05189999999966E-05	RNASEH2A	1.62436730732907E-12	<1E-12
SELENBP1	<1E-12	1.62447832963153E-12	RRM1	<1E-12	<1E-12

SELP	1.66533453693773E-15	3.67272878776248E-12	RRM2	1.62447832963153E-12	<1E-12
SEMA5A	1.62436730732907E-12	1.94655402907529E-12	RSRC1	<1E-12	<1E-12
SGCA	1.62447832963153E-12	<1E-12	SHMT2	<1E-12	1.62447832963153E-12
SGCG	<1E-12	1.62481139653892E-12	SKP2	<1E-12	<1E-12
SLC14A1	1.84450232865174E-11	1.535110E-03	SLC12A8	<1E-12	1.62470037423645E-12
SLC15A2	1.62447832963153E-12	1.62436730732907E-12	SLC6A8	1.62447832963153E-12	<1E-12
SLC19A3	1.62436730732907E-12	1.62447832963153E-12	SLC7A5	<1E-12	<1E-12
SLC1A1	<1E-12	1.62436730732907E-12	SMARCB1	1.62436730732907E-12	1.62436730732907E-12
SLIT3	1.62481139653892E-12	3.33066907387547E-16	SOX4	1.62447832963153E-12	<1E-12
SLPI	5.23000531771345E-11	1.62481139653892E-12	SPAG5	<1E-12	**1.62447832963153E-12**
SOX17	2.18119966532981E-11	1.64646074551911E-12	SPINT2	1.62447832963153E-12	<1E-12
SPARCL1	1.62447832963153E-12	1.0547118733939E-14	SPP1	<1E-12	1.62436730732907E-12
SRPX	3.11572989630804E-12	1.51419998761781E-09	SPRR1A	1.344130E-03	<1E-12
STARD13	1.62436730732907E-12	1.62447832963153E-12	SPTBN2	<1E-12	<1E-12
STARD8	1.4432899320127E-15	1.62447832963153E-12	SRM	<1E-12	<1E-12
STXBP6	1.62447832963153E-12	1.62725388719309E-12	SRPK1	1.62447832963153E-12	1.62436730732907E-12
SYNM	1.62447832963153E-12	1.62458935193399E-12	SSR4	<1E-12	1.62581059726108E-12
TAL1	1.62447832963153E-12	<1E-12	STMN1	1.62447832963153E-12	<1E-12
TBX4	<1E-12	1.62492241884138E-12	SULF1	1.62436730732907E-12	<1E-12
TCF21	1.62447832963153E-12	1.62447832963153E-12	SYT1	3.88145071639201E-12	1.02140518265514E-13
TMEM100	3.44013706410351E-12	4.65860683362962E-12	TFAP2C	<1E-12	<1E-12
TMEM47	1.62447832963153E-12	6.66133814775094E-16	THY1	<1E-12	8.70399999963745E-07
TNXB	4.44089209850063E-16	1.6974199823494E-12	TIMELESS	1.11022302462516E-16	1.62447832963153E-12
TPPP3	6.00399951977693E-10	3.33366667604196E-12	TK1	1.62447832963153E-12	<1E-12
TRHDE	<1E-12	1.11022302462516E-15	TMEM106C	<1E-12	1.6242562850266E-12
TRPC6	9.538300E-03	1.65156777143238E-12	TMEM132A	1.11022302462516E-16	1.62447832963153E-12
TSPAN12	1.62436730732907E-12	<1E-12	TMEM158	<1E-12	<1E-12
VIPR1	3.35698135955909E-12	2.15489848187644E-11	TMPRSS4	<1E-12	1.62447832963153E-12
VWF	7.43849426498855E-15	6.61679600000298E-11	TOP2A	1.62447832963153E-12	<1E-12
WFDC1	3.9190872769268E-14	<1E-12	TPX2	<1E-12	<1E-12
WISP2	1.13209441821027E-12	6.58073595616315E-12	TROAP	<1E-12	1.62436730732907E-12
ZBTB16	9.05719999000354E-09	1.05640000080953E-08	TTK	1.62447832963153E-12	1.62447832963153E-12
DPEP2	<1E-12	<1E-12	TUBG1	1.62447832963153E-12	1.62447832963153E-12
C1orf115	<1E-12	1.62458935193399E-12	TYMS	<1E-12	<1E-12
CAV2	1.62447832963153E-12	1.62447832963153E-12	UBE2C	<1E-12	<1E-12
COBL	1.62436730732907E-12	<1E-12	UCHL1	1.11022302462516E-16	<1E-12
CYP4B1	1.62447832963153E-12	1.62447832963153E-12	UCK2	<1E-12	<1E-12
ENPP4	7.7715611723761E-15	1.62436730732907E-12	UNG	1.62447832963153E-12	1.62447832963153E-12
FEZ1	1.62447832963153E-12	3.277000E-03	WDR12	1.62447832963153E-12	<1E-12
GPRASP1	2.81339995744645E-09	1.63036251166204E-12	ZWILCH	1.62436730732907E-12	<1E-12
HSPB6	<1E-12	1.62436730732907E-12	ZWINT	1.62447832963153E-12	<1E-12
ID4	1.62447832963153E-12	2.6274538100779E-12	CDCP1	<1E-12	1.11022302462516E-16
LAMP3	<1E-12	1.62447832963153E-12	COX6B1	<1E-12	<1E-12
LDB3	4.82960338388239E-11	2.8164004461928E-10	E2F8	1.62436730732907E-12	<1E-12

NPR3	7.74070000320037E-08	1.606450E-01	ENO1	1.62447832963153E-12	1.62447832963153E-12
RFX2	1.92210000027337E-07	1.37569999947296E-07	FUT3	1.62436730732907E-12	<1E-12
RGL1	2.19260165579271E-11	1.62447832963153E-12	ITGB4	1.62447832963153E-12	1.62447832963153E-12
RTN1	<1E-12	2.09920003602093E-09	LAD1	1.62447832963153E-12	<1E-12
SEC14L3	1.178090E-04	1.969320E-04	LRRC15	<1E-12	1.62436730732907E-12
TCEAL2	1.63369318073592E-12	1.17949983113874E-10	MDH2	1.62447832963153E-12	<1E-12
TTLL7	3.49389961407098E-10	3.02420000042325E-07	PLAU	1.62458935193399E-12	<1E-12
			RAB38	<1E-12	<1E-12
			SDC1	<1E-12	<1E-12
			SERPINH1	<1E-12	1.62447832963153E-12
			SHC1	1.62447832963153E-12	1.62436730732907E-12
			TMC5	1.62458935193399E-12	1.62447832963153E-12
