# Supplementary material for: Lung Cancer Gene Regulatory Network of Transcription Factors Related to the Hallmarks of Cancer
Source: Curr Issues Mol Biol. 2023 Jan 5;45(1):434–64. doi: 10.3390/cimb45010029 (PMC9857713; doi:10.3390/cimb45010029)
Supplement: Supplementary file 1 [file cimb-45-00029-s001.zip › Table S7.pdf]

NSCLC PAH

1. Fiber nodes: ABCA8, ACADL, ADRB1, ADRB2, AGTR1, AOX1, C1orf115, CA4,CAV1, CAV2, CD36, CDH5, CDO1, CHRDL1, CLDN18, CLIC5, CORO2B, CPA3,DUOX1, EMCN, FAM107A, FHL5, FMO2, GIPC2, GPM6A, HBB, LEPR, LIMS2,LRRC32, MMRN1, PALMD, PDK4, RASIP1, ROBO4, S1PR1, SELP, SGCG, SLC14A1,STXBP6, SYNM, TMEM100, TRHDE, TSPAN12, FERMT1, GPX2, TOP2A\\Regulator nodes: MNDA, EPAS1, ID4, KLF2, FOXF1, ZBTB16, TCF21\\
2. Fiber nodes: ACVRL1, PODXL, SERPINH1\\Regulator nodes: MNDA, EPAS1, KLF2, FOXF1, ZBTB16, TCF21\\
3. Fiber nodes: ASPA, DES, ASPM, CCNB1, DLGAP5, DTL, KIF11, NUSAP1, PLOD2,TK1, TYMS, ZWINT\\Regulator nodes: MNDA, EPAS1, ID4, KLF2, FOXF1, ZBTB16, TCF21, HLF,FOXM1\\
4. Fiber nodes: CFD, FBLN5, PDZD2, CEP55, LAD1, SLC7A5\\Regulator nodes: MNDA, EPAS1, ID4, KLF2, FOXF1, ZBTB16, TCF21, HLF\\
5. Fiber nodes: CLEC1A, GNG11, MMRN2, MYCT1, RAMP3, WISP2\\Regulator nodes: MNDA, EPAS1, ID4, KLF2, FOXF1, ZBTB16, TCF21, LMO2\\
6. Fiber nodes: ENPP4, SLC19A3\\Regulator nodes: MNDA, EPAS1, FOXF1, ZBTB16, TCF21, HOXC6\\
7. Fiber nodes: FEZ1, TPX2\\Regulator nodes: MNDA, EPAS1, ID4, KLF2, FOXF1, ZBTB16, TCF21, LMO2,HLF, FOXM1\\
8. Fiber nodes: FGR, RTN1\\Regulator nodes: MNDA, EPAS1, KLF2, FOXF1, ZBTB16, TCF21, LMO2, HOXC6\\
9. Fiber nodes: GPX3, NDC80\\Regulator nodes: MNDA, EPAS1, ID4, KLF2, FOXF1, ZBTB16, TCF21, FOXM1\\
10. Fiber nodes: HSPB6, PCOLCE2\\Regulator nodes: MNDA, ID4, KLF2, FOXF1, ZBTB16, TCF21\\
11. Fiber nodes: PTGER4, KIF20A\\Regulator nodes: MNDA, EPAS1, ID4, KLF2, FOXF1, ZBTB16, TCF21, LMO2,FOXM1, HOXC6\\
12. Fiber nodes: AURKA, BIRC5, CCNB2, ITGB4, MELK, NCAPG, PRC1\\Regulator nodes: MNDA, EPAS1, ID4, KLF2, FOXF1, ZBTB16, TCF21, LMO2, HLF, FOXM1, HOXC6\\
13. Fiber nodes: AURKB, BUB1, CENPE, KIF15, MCM4\\Regulator nodes: MNDA, EPAS1, ID4, KLF2, FOXF1, ZBTB16, TCF21, LMO2, HLF, FOXM1, SOX4, HOXC6, BZW2\\
14. Fiber nodes: BUB1B, RRM2\\Regulator nodes: MNDA, EPAS1, ID4, KLF2, FOXF1, ZBTB16, TCF21, HLF, FOXM1, HOXC6\\
15. Fiber nodes: CCNA2, E2F8, SKP2\\Regulator nodes: MNDA, EPAS1, KLF2, FOXF1, ZBTB16, TCF21, LMO2, HLF, FOXM1, SOX4, TFAP2C\\
16. Fiber nodes: CDC6, FEN1\\Regulator nodes: MNDA, EPAS1, ID4, KLF2, FOXF1, ZBTB16, TCF21, LMO2, HLF, FOXM1, SOX4, BZW2\\
17. Fiber nodes: DEPDC1, HJURP, KIF14, KIF2C, MCM2, MCM6, TIMELESS\\Regulator nodes: MNDA, EPAS1, ID4, KLF2, FOXF1, ZBTB16, TCF21, LMO2, HLF, FOXM1, SOX4\\
18. Fiber nodes: GINS1, HMMR\\Regulator nodes: MNDA, EPAS1, ID4, KLF2, FOXF1, ZBTB16, TCF21, HLF, HOXC6\\
19. Fiber nodes: NUF2, RAD51AP1\\Regulator nodes: MNDA, EPAS1, ID4, KLF2, FOXF1, ZBTB16, TCF21, HLF, FOXM1, HOXC6, TFAP2C\\
20. Fiber nodes: ADAMTS8, HIGD1B, VWF, SPP1, TMPRSS4\\Regulator nodes: EPAS1, ID4, KLF2, FOXF1, ZBTB16, TCF21\\
21. Fiber nodes: ADAMTSL3, AOC3, IGSF10, MFAP4\\Regulator nodes: EPAS1, ID4, KLF2, FOXF1, ZBTB16, TCF21, HLF\\
22. Fiber nodes: GPD1L, CDKN3\\Regulator nodes: EPAS1, ID4, KLF2, FOXF1, ZBTB16, TCF21, HLF, FOXM1\\

23. Fiber nodes: TRPC6, MAD2L1, SLC12A8\\Regulator nodes: EPAS1, ID4, KLF2, FOXF1, ZBTB16, TCF21, HLF, FOXM1,HOXC6\\
24. Fiber nodes: COL11A1, SULF1\\Regulator nodes: EPAS1, KLF2, FOXF1, ZBTB16, TCF21\\

NSCLC

1. Fiber nodes: ADH1B, AQP4, FABP4, IL33, LYVE1, PSAT1\\Regulator nodes: MNDA, EPAS1, ID4, KLF2, SOX17, NR4A3, ZBTB16, FOXF1, KLF4, TCF21\\
2. Fiber nodes: ANGPT1, FCN3, LDB2, MAMDC2, NPR3, PIP5K1B, SLC1A1, SRPX,TCEAL2\\Regulator nodes: MNDA, EPAS1, ID4, KLF2, SOX17, NR4A3, ZBTB16, FOXF1, KLF4, TCF21, GPRASP1\\
3. Fiber nodes: CXCL12, ITM2A, JAM2\\Regulator nodes: MNDA, EPAS1, ID4, KLF2, SOX17, NR4A3, ZBTB16, FOXF1, KLF4, TCF21, GPRASP1, LMO2\\
4. Fiber nodes: F8, CA9, NEK2\\Regulator nodes: MNDA, EPAS1, ID4, KLF2, SOX17, NR4A3, ZBTB16, FOXF1, KLF4, TCF21, GPRASP1, HLF, FOXM1\\
5. Fiber nodes: RGN, TTK\\Regulator nodes: MNDA, EPAS1, ID4, KLF2, SOX17, NR4A3, ZBTB16, FOXF1, KLF4, TCF21, GPRASP1, HLF, FOXM1, HOXC6\\
6. Fiber nodes: TMEM47, WFDC1\\Regulator nodes: MNDA, EPAS1, ID4, KLF2, SOX17, NR4A3, ZBTB16, FOXF1, KLF4, TCF21, GPRASP1, MEIS1\\
7. Fiber nodes: CCNE1, CENPF, PTTG1, RFC4, RNASEH2A, SPAG5\\Regulator nodes: MNDA, EPAS1, ID4, KLF2, SOX17, NR4A3, ZBTB16, FOXF1,KLF4, TCF21, GPRASP1, HLF, FOXM1, LMO2\\
8. Fiber nodes: ARHGAP6, FHL1, LAMP3, TNXB\\Regulator nodes: EPAS1, ID4, KLF2, SOX17, NR4A3, ZBTB16, FOXF1, KLF4,TCF21, GPRASP1\\
9. Fiber nodes: BMP5, PLAU\\Regulator nodes: EPAS1, ID4, KLF2, NR4A3, ZBTB16, FOXF1, KLF4, TCF21,GPRASP1, HLF\\
10. Fiber nodes: CA2, CYP4B1, SCGB1A1\\Regulator nodes: EPAS1, ID4, KLF2, SOX17, NR4A3, ZBTB16, FOXF1, KLF4,TCF21\\
11. Fiber nodes: ITGA8, PLCE1\\Regulator nodes: EPAS1, ID4, KLF2, SOX17, NR4A3, ZBTB16, FOXF1, KLF4,TCF21, GPRASP1, MEIS1\\

SCLC PAH

1. Fiber nodes: ABCA8, ACVRL1, CLEC1A, DUOX1, HSPB6, LIMS2, LRRC32, MYH10,PLLP, RAMP3, ROBO4, SLC19A3, TMC5, TPPP3, CCN5, NIPSNAP1, THY1\\Regulator nodes: TCF21, FOXF1, NR2F1, ZBTB16, EPAS1, KLF2, MNDA\\
2. Fiber nodes: ADRB2, AOC3, EMCN, FEZ1, GPD1L, SEMA5A, SLIT3\\Regulator nodes: TCF21, FOXF1, NR2F1, ZBTB16, EPAS1, KLF2, MNDA, SOX4\\
3. Fiber nodes: CAV1, CD36, GNG11, IGSF10, MMRN1, PLAC8, ENO2, PRPF19\\Regulator nodes: TCF21, FOXF1, NR2F1, EPAS1, KLF2, MNDA\\
4. Fiber nodes: CDH5, TSPAN12\\Regulator nodes: TCF21, FOXF1, NR2F1, EPAS1, KLF2, MNDA, ID4\\
5. Fiber nodes: DPEP2, FGR, FKBP4\\Regulator nodes: TCF21, FOXF1, NR2F1, ZBTB16, EPAS1, KLF2, MNDA, ID4\\
6. Fiber nodes: RASIP1, SPARCL1\\Regulator nodes: TCF21, FOXF1, NR2F1, EPAS1, KLF2, MNDA, LMO2\\
7. Fiber nodes: ADAMTS8, CAV2, CPA3, DENND2A, PTGER4\\Regulator nodes: FOXF1, NR2F1, EPAS1, KLF2, MNDA\\
8. Fiber nodes: CA4, ESPL1\\Regulator nodes: FOXF1, EPAS1, KLF2, MNDA, ID4, LMO2\\
9. Fiber nodes: CLIC5, FMO2\\Regulator nodes: FOXF1, EPAS1, KLF2, MNDA\\
10. Fiber nodes: HIGD1B, GPX2\\Regulator nodes: FOXF1, ZBTB16, EPAS1, KLF2, MNDA\\
11. Fiber nodes: PDE5A, VWF\\Regulator nodes: FOXF1, NR2F1, EPAS1, KLF2, MNDA, LMO2\\

12. Fiber nodes: AURKA, CCNB1, CENPA\\Regulator nodes: FOXF1, ZBTB16, EPAS1, KLF2, MNDA, HOXC6, SOX4, HLF\\
13. Fiber nodes: BUB1B, KIF11, KIF2C, TROAP\\Regulator nodes: FOXF1, ZBTB16, EPAS1, KLF2, MNDA, FOXM1, HOXC6, SOX4,HLF\\
14. Fiber nodes: CCNF, SRPK1\\Regulator nodes: FOXF1, NR2F1, ZBTB16, EPAS1, KLF2, MNDA, FOXM1, HOXC6, SOX4, ID4, HLF\\
15. Fiber nodes: CDKN3, HMMR, RAD51AP1\\Regulator nodes: FOXF1, NR2F1, ZBTB16, EPAS1, KLF2, MNDA, HOXC6, SOX4, HLF\\
16. Fiber nodes: CHAF1A, RFWD3\\Regulator nodes: FOXF1, NR2F1, ZBTB16, EPAS1, KLF2, MNDA, FOXM1, HOXC6, SOX4, HLF\\
17. Fiber nodes: ACTL6A, CEP55\\Regulator nodes: NR2F1, ZBTB16, MNDA, FOXM1, HOXC6, SOX4, HLF\\
18. Fiber nodes: ASF1B, BUB1, CDCA4, CENPM, NUF2, RRM2\\Regulator nodes: NR2F1, ZBTB16, FOXM1, HOXC6, SOX4, HLF\\
19. Fiber nodes: CFD, CLDN18, PCOLCE2, TMEM100, RTN1\\Regulator nodes: NR2F1, EPAS1, KLF2, MNDA\\
20. Fiber nodes: CHEK1, RAD54L\\Regulator nodes: NR2F1, ZBTB16, KLF2, FOXM1, HOXC6, SOX4, HLF\\
21. Fiber nodes: PDK4, COL5A2, TOP2A\\Regulator nodes: ZBTB16, SOX4\\
22. Fiber nodes: TRPC6, DBN1\\Regulator nodes: ZBTB16, KLF2, MNDA, LMO2\\
23. Fiber nodes: ASPM, CCNB2, SULF1\\Regulator nodes: ZBTB16, MNDA, SOX4, HLF\\
24. Fiber nodes: CCNA2, MCM2\\Regulator nodes: ZBTB16, FOXM1, HOXC6, SOX4, HLF\\
25. Fiber nodes: DEPDC1, MCM4\\Regulator nodes: ZBTB16, EPAS1, KLF2, FOXM1, HOXC6, SOX4, HLF\\
26. Fiber nodes: ENO1, MCM7, TUBG1\\Regulator nodes: ZBTB16, FOXM1, HOXC6, SOX4, LMO2, HLF\\
27. Fiber nodes: NUSAP1, SPP1\\Regulator nodes: ZBTB16, MNDA, SOX4\\
28. Fiber nodes: ACTG2, MGP\\Regulator nodes: EPAS1, KLF2, MNDA\\
29. Fiber nodes: SYT1, UCHL1\\Regulator nodes: EPAS1, KLF2, MNDA, ID4, LMO2\\

SCLC

1. Fiber nodes: ADH1B, JAM2, LTBP4, WFDC1, MMP11, PPAT\\Regulator nodes: TCF21, FOXF1, ZBTB16, NR2F1, EPAS1, KLF2, MNDA, SOX4,DLX5\\
2. Fiber nodes: ANGPT1, CYB5A, DUSP1, CLXN, ITM2A, LDB2, MAMDC2, PZP\\Regulator nodes: TCF21, FOXF1, NR2F1, EPAS1, KLF2, MNDA, DLX5\\
3. Fiber nodes: AQP4, CXCL12, ENG, GIMAP6, GYPC, IL33, ITGA8, MYL9,SELENBP1, SLC1A1, SRPX, TMEM47\\Regulator nodes: TCF21, FOXF1, ZBTB16, NR2F1, EPAS1, KLF2, MNDA, DLX5\\
4. Fiber nodes: FGL2, FHL1, FZD4\\Regulator nodes: TCF21, FOXF1, ZBTB16, NR2F1, EPAS1, KLF2, MNDA, DLX5, ID4\\
5. Fiber nodes: PDE8B, PTTG1\\Regulator nodes: ZBTB16, MNDA, SOX4, DLX5, LMO2, HLF\\
6. Fiber nodes: CDCA3, CDCA8, LMNB2, SPAG5\\Regulator nodes: ZBTB16, EPAS1, KLF2, MNDA, SOX4, DLX5, FOXM1, HOXC6, HLF\\
7. Fiber nodes: FANCI, KIF4A\\Regulator nodes: ZBTB16, NR2F1, EPAS1, KLF2, MNDA, SOX4, DLX5, FOXM1, HOXC6, HLF\\
8. Fiber nodes: MCM10, OIP5\\Regulator nodes: ZBTB16, EPAS1, KLF2, SOX4, DLX5, FOXM1, HOXC6, HLF, RFX2\\
9. Fiber nodes: FABP4, SCGB1A1\\Regulator nodes: EPAS1, KLF2, MNDA, DLX5\\
